# Advances in Quantitative Imaging of Genetic and Acquired Myopathies: Clinical Applications and Perspectives

**DOI:** 10.3389/fneur.2019.00078

**Published:** 2019-02-11

**Authors:** Matteo Paoletti, Anna Pichiecchio, Stefano Cotti Piccinelli, Giorgio Tasca, Angela L. Berardinelli, Alessandro Padovani, Massimiliano Filosto

**Affiliations:** ^1^Neuroradiology Department, IRCCS Mondino Foundation, Pavia, Italy; ^2^Department of Brain and Behavioural Sciences, University of Pavia, Pavia, Italy; ^3^Unit of Neurology, Center for Neuromuscular Diseases, ASST Spedali Civili and University of Brescia, Brescia, Italy; ^4^Neurology Department, Dipartimento di Scienze dell'Invecchiamento, Neurologiche, Ortopediche e della Testa-Collo, Fondazione Policlinico Universitario A. Gemelli IRCCS, Rome, Italy; ^5^Child Neurology Unit, IRCCS Mondino Foundation, Pavia, Italy

**Keywords:** muscular imaging, neuromuscular diseases, MRI, myopathy, advanced imaging

## Abstract

In the last years, magnetic resonance imaging (MRI) has become fundamental for the diagnosis and monitoring of myopathies given its ability to show the severity and distribution of pathology, to identify specific patterns of damage distribution and to properly interpret a number of genetic variants. The advances in MR techniques and post-processing software solutions have greatly expanded the potential to assess pathological changes in muscle diseases, and more specifically of myopathies; a number of features can be studied and quantified, ranging from composition, architecture, mechanical properties, perfusion, and function, leading to what is known as quantitative MRI (qMRI). Such techniques can effectively provide a variety of information beyond what can be seen and assessed by conventional MR imaging; their development and application in clinical practice can play an important role in the diagnostic process and in assessing disease course and treatment response. In this review, we briefly discuss the current role of muscle MRI in diagnosing muscle diseases and describe in detail the potential and perspectives of the application of advanced qMRI techniques in this field.

## Introduction

The term “myopathies” encompasses a wide spectrum of childhoodand adulthood, acquired and inherited diseases affecting skeletal muscle whose main feature is muscle weakness. Other features that may be variably associated are muscle pain, contractures, exercise intolerance, myoglobinuria, and multi system involvement i.e., heart, liver, central, and peripheral nervous system (1).

The diagnosis of myopathy has been traditionally based on the clinical, electromyography and muscle biopsy findings, which indicated the subsequent genetic investigations.

Currently, however, there is a large availability of other powerful diagnostic technologies, especially in the field of genetics, which have widely changed our approach to the diagnosis of muscle diseases. The possibility to broadly study patients' genomes through Next Generation Sequencing (NGS), approaches such as Whole Exome Sequencing (WES) and Whole Genome Sequencing (WGS) are already revolutionizing the entire diagnostic process, allowing us to detect several molecular changes whose role and pathogenicity need to be evaluated in the specific clinical context (2).

In the last years, magnetic resonance imaging (MRI) has become a very powerful tool to improve our capability of diagnosing muscle diseases because it can show the severity and distribution of tissue damage in different muscles of the body or within a single muscle. The resulting observations are an extremely useful complement to clinical evaluation, especially when specific patterns of damage distribution are identified, and help in reaching the correct diagnosis and the proper interpretation of the identified genetic variants (3–5).

In this review, we briefly discuss the current role of muscle MRI in diagnosing muscle diseases and describe in detail the advances and the perspectives of the application of advanced quantitative MRI (qMRI) techniques in this field.

## Conventional Muscle Imaging

MRI techniques used in muscle disorders have traditionally included T1-weighted images (T1w) and sequences sensitive to tissue water, as short-tau inversion recovery (STIR) or T2-weighted images (T2w), with or without suppression of the signal of fat tissue. The use of such techniques has been primarily devoted to the macroscopic evaluation of morphological changes of the muscles, including fat replacement and edema.

Aiming to more precisely grade the compositional changes in muscle, semi-quantitative indices have been implemented. In clinical and research practice, in fact, several MRI rating scales [usually comprising 4 or 5 grades ranging from normality (0) to severe abnormalities (4, 5)] are currently used to evaluate the extension of fatty degeneration and semi-quantitatively assess the degree of muscle damage, the most used being the Mercuri and Fischer scales (3, 4).

In the recent years, a part of literature in the field of muscle disorders evaluated through comparative studies the distribution and severity of damage in different muscles through comparative studies in order to detect specific MRI patterns for different myopathies, helping the clinicians in the diagnostic workup.

Characteristics of muscle damage on conventional MRI may be organized into the following main pattern descriptors: (1) severity and distribution of morphological changes in muscle size and shape, (2) severity and distribution of fat, and (3) presence of muscle edema (5).

Among acquired myopathies, the idiopathic inflammatory myopathies (IIM) are certainly the best characterized. In this group of diseases, MRI can visualize edema (as a sign of inflammatory changes) on short tau inversion recovery (STIR) or fat-suppressed T2-weighted sequences in the early/acute disease phases, whereas fatty replacement and muscle atrophy on T1-weighted images can occur as later changes (5, 6).

In Polymyositis (PM), muscle edema usually affects proximal muscles of the lower and upper extremities in a symmetrical way (6). Compared with PM, Dermatomyositis (DM) affects predominantly and more severely the thigh muscles, except those of the posterior compartment, which are similarly involved in both disorders (7).

In 2014, Miranda et al. compared MRI images of thigh muscle compartments of 11 adult subjects with DM and 11 adult subjects with PM (8). Muscle edema, mainly in the proximal region of the studied muscles, was significant in DM, whereas areas of fat replacement were predominant in PM.

In 2011, Davis et al. proposed a specific scoring system for assessing acute inflammatory changes in juvenile DM (9). Markers of inflammatory change in four muscle groups and the surrounding soft tissues were defined: degree of muscle inflammation based on a 4-point score (none = 0, mild = 1, moderate = 2, and severe = 3), assessed by taking the overall impression of the entire muscle group; presence of soft-tissue edema (absent = 0, present = 1) and of perifascicular edema (absent = 0, present = 1).

In sporadic Inclusion Body Myositis (sIBM), a myodegenerative and inflammatory muscle disease, muscle atrophy is at least as frequently detected as inflammation on MRI, predominantly affecting the distal quadriceps with a pattern of involvement reported in retrospective studies as sensitive and specific for the diagnosis (10).

Inherited myopathies have been studied in depth by muscle MRI and several specific patterns have been identified.

In 50 genetically confirmed Duchenne Muscular Dystrophy (DMD) children, muscle MR and qualitative assessment by Mercuri scoring showed severe fibro-fatty changes in gluteus medius, minimus, and adductor magnus (11). Moderate to severe changes in gluteus maximus and quadriceps muscles were also observed whereas sartorius, semimembranosus and gracilis were spared. Superficial posterior and lateral leg muscles were preferentially involved with sparing of deep posterior and anterior leg muscles. A direct correlation between duration of illness and fibro-fatty changes in piriformis, quadriceps, and superficial posterior leg muscles was also observed (11).

In 51 molecularly confirmed Becker Muscular Dystrophy (BMD) patients, muscle MRI showed a pattern of fatty substitution involving mainly the hip extensors and most thigh muscles (12). The severity of muscle fatty substitution significantly correlated with specific DMD gene mutations (i.e., patients harboring deletion of exon 48 or deletions bordering exon 51 presented a milder involvement). Skeletal muscle MRI also correlated with motor function and helped in predicting functional deterioration within a 12-months period.

Limb-Girdle Muscular Dystrophies (LGMD) are a molecularly heterogeneous group of childhood or adult-onset muscular dystrophies with frequent clinical overlap that often causes considerable diagnostic difficulty. In LGMD R1 (former LGMD2A), caused by mutations in the calpain-3 gene, MRI pattern shows involvement of the posterior thigh and adductor muscles (13). At lower leg level, MRI usually detects involvement of the soleus and of the medial head of the gastrocnemius with relative sparing of the lateral head (13). LGMD R9 (former LGMD2I), caused by mutations in fukutin-related protein (FKRP), is characterized by MRI signal changes in adductor muscles and in posterior thigh and posterior thigh and calf muscles, whereas tibialis anterior is usually spared and often hypertrophied. During the disease course, gluteus maximus, adductor magnus, and biceps femoris are commonly involved while vastus lateralis and vastus intermedius muscles can be spared until advanced stage of disease (14).

The group of dysferlinopathies, caused by mutations in the dysferlin gene, includes the proximal form LGMD R2 (former LGMD2B), the distal Miyoshi myopathy, clinically characterized by initial calf involvement, and a form of distal anterior (tibial) myopathy.

Despite clinical heterogeneity, these myopathies are characterized by a common MRI pattern in the lower limbs, with involvement of gastrocnemius medialis, soleus, posterior thigh, and adductor compartments. While anterior compartments of the thighs may also be involved, there is usually a characteristic sparing of the gracilis and sartorius muscles in the thigh and, importantly, of the pelvic muscles compared to the thigh and lower legs (14–16).

At variance, sarcoglycanopathies (LGMD R3-R6, former LGMD2C-2F) are characterized by relative or even complete sparing of the lower leg compared to the thigh, and a clear proximo-distal involvement of the vastus lateralis. This pattern is consistent across the four different mutated sarcoglycans (alpha, beta, gamma, and delta) (17).

Among the other inherited myopathies, autosomal dominant myopathies such as LGMD D1 (former LGMD1D), facio-scapulo-humeral muscular dystrophy (FSHD), myofibrillar myopathies (MFM), and some forms of congenital myopathies were the better studied.

In LGMD D1, a peculiar “horseshoe sign” given by fatty replacement of posterior thigh and adductor magnus muscles with sparing of the semitendinosus is the MRI hallmark of most of the mutated patients, together with gastrocnemius medialis and later on soleus involvement at the lower leg level (18).

In FSHD, the involvement in the lower limbs is more variable although some muscles are consistently affected (such as abdominal muscles, semimembranosus and other muscles of the posterior thigh, and gluteus minimus) and others consistently spared even at advanced stages, such as iliopsoas and obturator muscles (19).

MFMs, pathologically characterized by myofibrillar degeneration and accumulation of abnormal proteins such as desmin, alpha-B-Crystallin, and Myotilin, are a group of diseases often difficult to diagnose. In this setting, muscle imaging, in combination with clinical and muscle biopsy evaluation, can be a helpful tool in identifying the specific MFM subtypes (4).

In patients with desminopathy, the semitendinosus was the most affected muscle, followed by sartorius and gracilis, whereas other thigh muscles were involved later in the disease course. At the leg level, the peroneal muscles were more, or at least equally, involved than tibialis anterior muscle, which is considered a criterium to detect desminopathy with sensitivity of 100% and specificity of 95% among MFMs (4).

MFMs having mutations in *MYOT, FLN-C*, and *LDB3* (ZASP*)* showed different patterns of muscle degeneration, which led to the formulation of criteria useful for separation of distinct MFM subtypes and in directing genetic analysis (20).

Central Core Disease (CCD) is caused by mutations in the ryanodine receptor 1 gene (RYR1). In the autosomal dominant form, muscle MRI shows selective involvement of vasti, sartorius, adductor magnus with relative sparing of rectus femoris, gracilis, and adductor longus in the thigh and selective involvement of soleus, gastrocnemii, and peroneal group with relative sparing of the tibialis anterior in the leg (21).

Muscle MRI performed in a cohort of patients with muscle disorders characterized by rigidity of the spine (rigid spine syndrome *SEPN1*-related, Bethlem myopathy, and Ullrich congenital muscular dystrophy linked to *Col6A1, Col6A2*, and *Col6A3* mutations, Emery-Dreifuss muscular dystrophy linked to *LMNA* defects and calpain-deficient limb girdle muscular dystrophy) showed a pattern typical for 1 of the 5 studied forms in 82% of the scans and a pattern consistent, although not typical, in 8% of the cases (22).

A selective MRI pattern was recognized in selenoprotein N (SEPN1)-related myopathies. The semimembranosus muscle is always largely involved to large extents up to a complete atrophy, often together with sartorius, adductor magnus, and the short head of biceps femoris, whereas abnormalities of the lower leg are less common. in the other districts of whole-body scans, severe wasting of sternocleidomastoid muscle was detected whereas elevator scapulae in the neck, iliacus in the pelvis, adductor longus, gracilis, rectus femoris, and tibialis anterior in the lower limb are spared even in later stages (23).

Within the metabolic myopathy group, Pompe disease is the best-characterized disease, particularly for what concerns the late onset form (LOPD). A systematic axial muscle degeneration, especially in spine extensor and abdominal muscles and thigh muscles, is often recognizable (24, 25), with relative sparing of leg muscles. The scapular girdle and pelvic girdle muscles were also involved and a distinctive feature can be the tongue muscle fatty degeneration, evident in most of affected patients (26).

**Figure 1:**
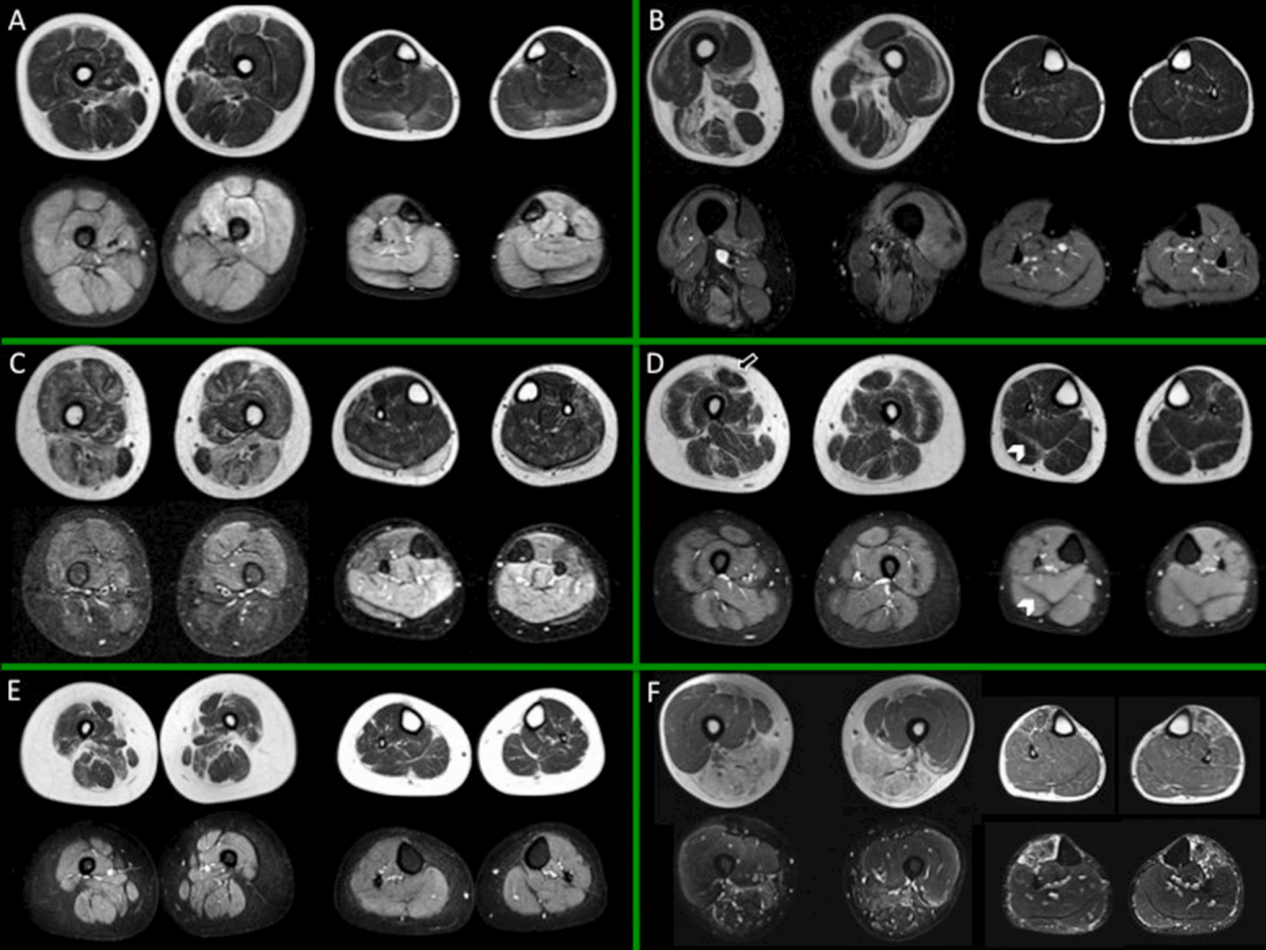
Muscle MRI in a subset of dystrophies and congenital myopathies. For each box, thighs are displayed on the left, legs on the right side; spin-echo T1-weighted images are on top, short-tau inversion recovery (STIR) images on the bottom. **(A)** Child with Duchenne dystrophy (DMD) showing prominent fat replacement of gemini and adductor magnus and, to a lesser extent, of soleus, peroneal muscles, and rectus femoris bilaterally. Of note, edema of the left quadriceps and, more diffuse, of the leg muscles is evident. **(B)** Adult subject with Pompe Disease, showing selective fatty replacement of the posterior thigh and relative sparing of leg muscles. Slight patchy STIR hyperintensities are evident bilaterally, mainly involving the thigh musculature, as reported in literature (58). **(C)** Young subject with beta sarcoglycanopathy, showing important involvement of adductor magnus and longus and harmstring; at leg level the extensor digitorum longus is the most affected muscle. Unspecific slight STIR hyperintensity of the residual calf muscles is evident. **(D)** Young subject with Bethlem myopathy showing predominantly peripheral involvement both at the level of the thigh and leg muscles. Note the central shadow in the rectus femoris (arrow) and the bat-wing sign in the posterior leg musculature (arrowheads). No edema is evident. **(E)** Young subject with congenital myopathy due to selenoprotein 1 (SEPN1) showing diffuse muscular atrophy and selective fatty replacement of sartorius and, to a lesser extent, of adductor magnus; no edema is evident. **(F)** Young subject with facio-scapulo-humeral dystrophy (FSHD), showing involvement of the posterior thigh muscles and of tibialis anterior (TA), with sparing of the quadriceps bilaterally, but with rectus femoris involvement. Edema is evident at the level of TA and harmstring muscles and, peripherally, of vastus lateralis. All presented MRI images have been collected during routine patient care.

**Figure 2:**
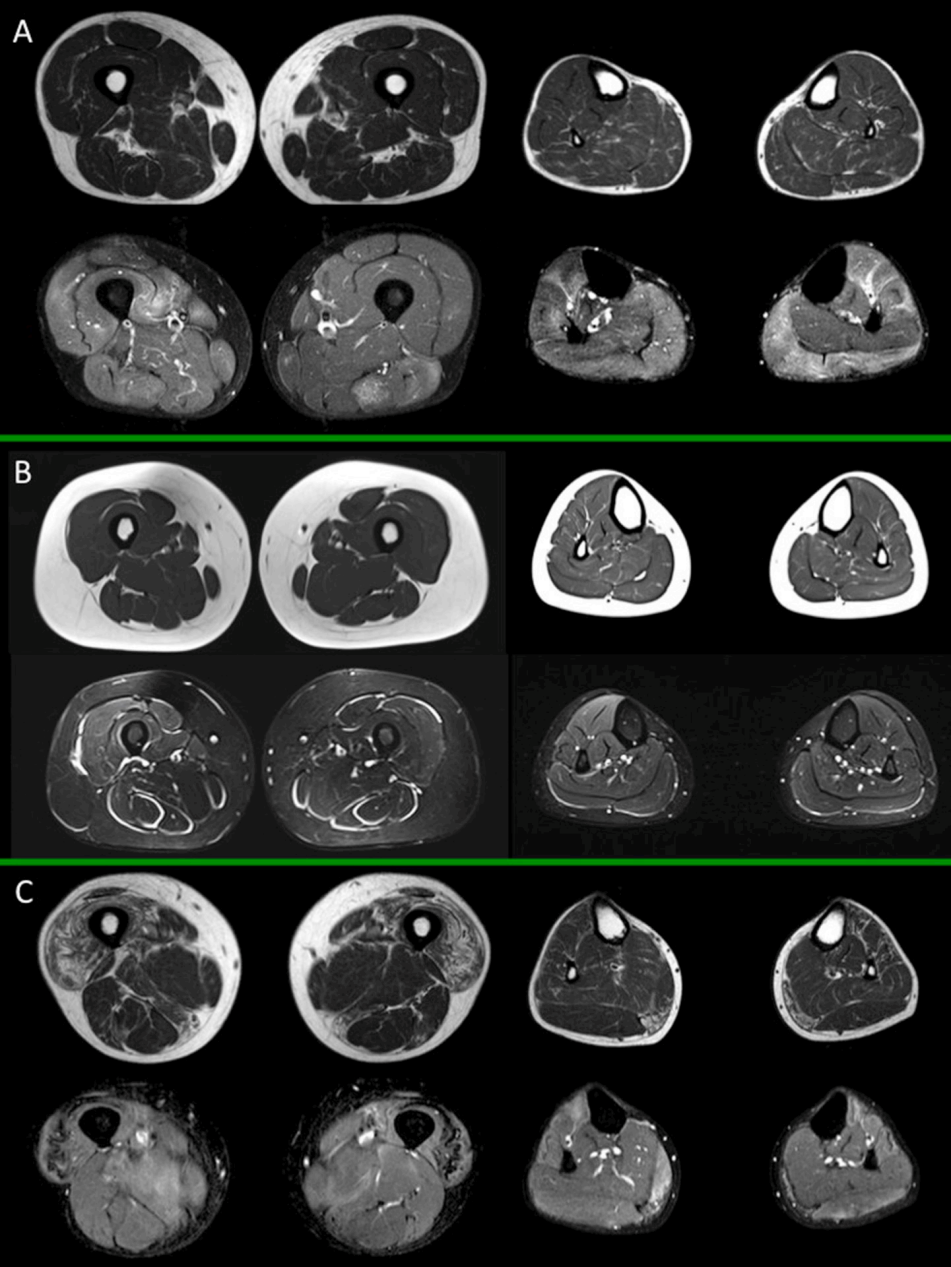
Muscle MRI in inflammatory myopathies. For each box, thighs are displayed on the left, legs on the right side; spin-echo T1-weighted images are on top, short-tau inversion recovery (STIR) images on the bottom. **(A)** Adult subject with polymyositis (PM), showing multifocal muscular edema involving both thigh and leg muscles and no selective pattern of fat replacement; **(B)** Adult subject with dermatomyositis (DM) showing characteristic multifocal perifascicular edema involving both thigh and leg muscles; **(C)** Adult subject with inclusion body myositis (IBM), with predominant fatty substitution of anterior thigh musculature and gemini muscles, associated to slight multifocal muscle edema. All presented MRI images have been collected during routine patient care.

In Figures 1, 2 different patterns of involvement of both hereditary and inflammatory myopathies are shown.

## Applications of Advanced Quantitative Techniques

The advances in MRI techniques and post-processing software solutions have greatly expanded the potential to assess pathologic changes in muscle disorders; a number of features can be studied and quantified, ranging from composition, architecture, mechanical properties, perfusion, and function, leading to what is known as quantitative MRI (qMRI).

Such techniques can effectively provide information beyond what can be seen and assessed at conventional MRI; their development and application in clinical practice can play an important role in defining the disease course and treatment response (27–30).

### Fat Fraction

The calculation of the percentage of fat into a tissue, called fat fraction (FF), has become a crucial point in the study of myopathies, where fat replacement is an important feature of the pathological spectrum (31).

Dixon imaging (developed by Thomas Dixon in 1984) is an MR technique that relies on water/fat chemical shift difference and that is used to obtain homogeneous fat-suppression when other techniques fail (mainly due to inhomogeneities of the magnetic field) and to calculate the FF of a tissue (32–35).

**Figure 3:**
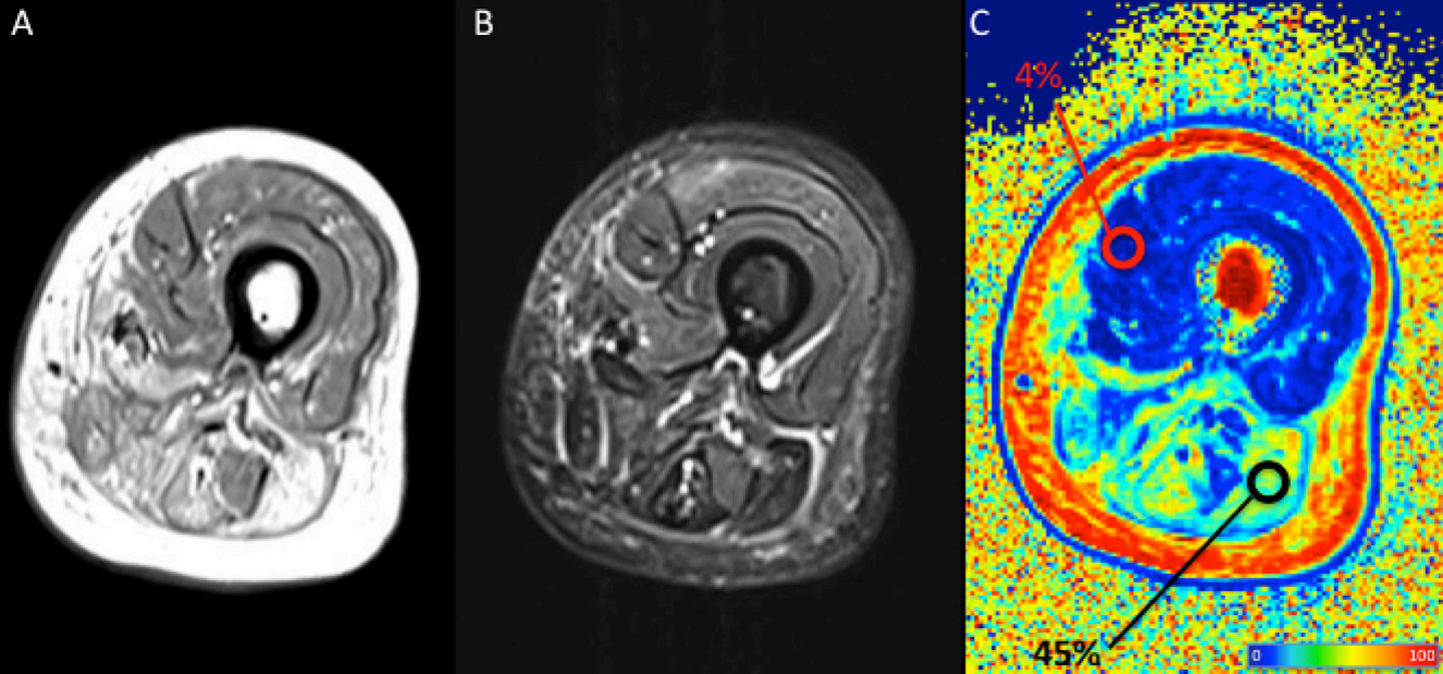
Qualitative and quantitative left thigh muscle MRI in an adult subject with facio-scapulo-humeral disease (FSHD). **(A)** T1-weighted, **(B)** short-tau inversion recovery (STIR), and **(C)** fat fraction (FF) calculated with a 3-point Dixon approach are displayed. Regions-of-interest (ROI) are positioned, respectively on normal and fatty replaced muscles, with their mean FF shown as percentage in the tag. The figure shows extensive fatty replacement of the harmstring muscles (mean FF = 4%), with the ROI positioned on the long head of the biceps femoris, compared to the normal quadriceps (mean FF = 45%). Colors from dark blue to dark red represent the percentage of fat (FF) (see color bar). MRI images have been collected during routine patient care.

The Dixon sequence basically acquires two images, one with water and fat signals in-phase and the other with water and fat signals out-of-phase: the sum and subtraction of the two images allows the production of a water-only image and a fat-only image, the latter being of particular interest in muscle imaging. A FF map is usually built with a colorimetric scale based on the percentage of fat in each voxel; regions of interest (ROI) can be drawn to calculate the FF in a chosen area within the muscle belly (Figure 3). The FF as measured by Dixon imaging is considered highly reliably and reproducible (36–38). A non-uniform fat replacement throughout the muscle body, however, has to be taken into account: Hooijmans et al. recently found that distribution of fat replacement in DMD varies along the proximodistal axis, with higher FF in the distal and proximal muscle segments compared to the belly. As a consequence such non-uniformity have to be taken into account to prevent sample bias (39).

Different techniques are available for Dixon imaging. Two-point Dixon is generally considered satisfactory for the liver; artifacts as image swap may occur, however, in skeletal muscle. Three-point Dixon, a technical more reliable evolution of the original sequence, is almost always not affected by such an issue (40).

Quantitative Dixon MRI is more precise than visual radiological methods as semi-quantitative grading for the evaluation of fat replacement, which commonly implies a systematic overestimation of the fatty component (41, 42). The use of Dixon-derived FF, therefore, has become a reference for a precise assessment of fat replacement in muscular dystrophies, especially in DMD, where it has been more frequently applied. In DMD, in fact, FF maps correlate well with histology (37, 43), play a role in the follow-up (44–47) and in the evaluation of therapy response (48).

The quantification of FF also correlates well with clinical function (49, 50); a threshold of 50% of fat replacement, as assessed by Dixon imaging, can predict the loss of ambulation with a sensitivity/specificity of 100%/91% in DMD (50). Hence FF evaluation thus represents a promising endpoint to test the efficacy of treatment over the degree of ambulation impairment.

As aforementioned, Dixon-derived FF estimation has revealed its usefulness in monitoring the effects of treatments in DMD. Arpan et al. showed that 1 year administration of corticosteroids to young DMD boys stopped the fatty infiltration process in the thighs and legs, whereas in the natural course of the disease fatty infiltration rate was, respectively, of 7 and 3% (48): the authors suggested that the lesser fat deposition in steroid therapy may be explained by a reduced inflammation/damage and fat infiltration secondary to treatment. Janssen et al. used T2-derived FF to monitor and quantify the therapeutic effects of physical activity in FSHD subjects, revealing a slowing of the fat replacement rate from the usual care group, and also demonstrated that FF can be a reproducible and sensitive biomarker (51).

Dixon-based FF has been also used to quantify fatty muscle degeneration and track disease progression in Becker muscular dystrophy (BMD), even if in a small cohort and at a relatively short imaging interval of follow-up (52). Fischer et al. documented that mean FF (and T2 measure) were highly negatively correlated with time function tests in 20 BMD subjects, suggesting that the mean FF (and, in parallel, T2) are able to measure the functional impact of key pathological processes (53).

Subtle changes in FF have been shown in oculopharyngeal muscular dystrophy (OPMD) (54) and in in LGMD R9, where an increase in fat replacement was demonstrated in 9 (out of 14) muscles analyzed over a 12 months follow-up, whereas such a changement was not evident on semi-quantitative scoring of the T1w images (55).

FF quantification sensitively detected fat replacement rate also in Charcot-Marie-Tooth disease 1A and 2F (CMT1A-2F) and sIBM (56–58).

In Pompe disease authors showed a strong correlation between muscle strength, muscle functional scales and the degree of intramuscular fat as assessed by a three-point Dixon sequence (59). Carlier et al. retrospectively demonstrated with Dixon imaging that fat replacement occurs at a rate of 0.9%/year in Pompe disease, hamstring and adductor muscles being the most rapidly involved (60). More recently, in a prospective study in 32 late onset Pompe disease (LOPD) subjects, Figueroa-Bonaparte et al. demonstrated a 1.7% increase in fat content of thigh muscles at 1 year follow-up, but only in symptomatic subjects (61).

As a further implementation of the Dixon technique, a T2w TSE Dixon approach was used by Schlaeger et al. (62) as a fast whole-body simultaneous grading of muscle fat replacement and edema in a mixed cohort of subjects with neuromuscular disorders. The authors documented a high inter-method agreement between traditional T1w Dixon, T2 STIR, and the proposed T2 TSE Dixon for rating fatty infiltration and intramuscular edema, with no need for multiple acquisition stacks to cover the entire body and the possibility to study contemporarily fat replacement and edema. As a consequence, T2-weighted Dixon TSE imaging was proposed as a further step in qMRI to obtain an accelerated simultaneous grading of whole-body skeletal muscle fat infiltration and edema (62).

With regard to magnetic field inhomogeneities that affect Dixon sequences, a series of modifications of the initial Dixon technique have been proposed to reduce this issue leading first to the three-point Dixon and later to the so called iterative decomposition of water and fat with echo asymmetry and least-square estimation (IDEAL) (63). Such technique is essentially compatible with any pulse sequence.

The major strengths of this technique are the uniform and reliable fat suppression throughout the body and the very high signal to noise ratio (SNR) of the reconstructed fat-only and water-only (64). The purpose and the utilization of such technique, however, remains the same as the two- and three-point Dixon.

In conclusion, the quantification of fat-fraction measurement as obtained from Dixon (or even IDEAL) MRI can be regarded as a useful biomarker to precisely quantify the fatty infiltration in muscle (Figure 3), representing a marker of involvement (though relatively-late), and a marker of therapeutic response (31).

### Relaxometry

#### T2 Relaxometry

T2 mapping is a qMRI technique that measures T2 relaxation of tissues, that reflects the locoregional water binding and that is prolonged by the presence of increased free water (20, 65–67). The T2 time is characteristic for each tissue and is affected by a number of physiologic and pathologic processes; in muscle imaging it has been used to explore the effects of exercise (65, 68, 69), and to specifically evaluate neuromuscular diseases, also after exercise (70–74). It must be underlined that, given known modification of T2 relaxation time after exercise, performing an MRI shortly after physical activity may significantly alter the signal; an appropriate resting time is therefore recommended.

T2 measurement is an attractive endpoint because it is primarily affected by early changes in muscle integrity, before fat infiltration occurs: in other words, the T2 value reflects the prodromal phases of the disease in the muscle. As a consequence, the T2 is an *in vivo* indicator of ongoing disease activity. Nonetheless its low specificity has to be kept in mind: an increase of T2 signal may be due to several unspecific events which all lead to intracellular or extracellular edema or a combination of both (29).

The difference between global T2 and water T2 must be specified. Global T2 signal is strongly influenced by fat infiltration, which is better described by fat-fraction evaluation techniques as Dixon (see above); the use of global T2 can thus lead to confusion and is therefore discouraged. What is really interesting in the setting of muscle diseases imaging is water T2, that reflects the ongoing pathologic changes in the muscle (75).

In DMD T2 was found to be increased in involved muscles in a series of studies (71, 76–82). Moreover, T2 and related variables were shown to increase over time and to strongly correlate with changes in functional performance (83, 84).

Studies combining qMRI and magnetic resonance spectroscopy (MRS) in DMD demonstrated interesting findings in the early detection of muscle involvement and in the assessment of steroid therapy effects. Hooijmans et al. showed that both T2 and PDE (phosphodiesters, see ^31^P-MRS section below) are already increased prior to fat replacement and remain elevated in affected muscles. Both measures could therefore serve not only as early markers of disease, but they could also potentially track response to therapy (85).

As a proof of principle, Arpan et al. in fact, demonstrated, through a combined quantitative MRI/MRS approach, that both quantitative MR techniques are able to effectively monitor the effects of corticosteroids on the lower extremity muscles of young boys with DMD. In treated subjects, T2 values and FF decreased when compared to untreated boys, and progressive fat replacement was slower during treatment, at least in a few muscles. The monitoring of T2 was able to detect therapeutic effects as early as 3 months after drug initiation (48).

Differently from DMD, BMD subjects did not show a T2 increase in affected muscles (86), likely in keeping with the minor degree of inflammation. Conversely, high T2 values were found in residual muscle tissue of myotonic dystrophy (DM) 1, with the highest values reported in the more advanced stages of disease, the increase being associated to a decrease in muscle force (72).

Finally, increased T2 values were also found in inflammatory myopathies, where they again reflect the degree of inflammation (58, 73, 87, 88).

#### T1 Relaxometry

Another available technique based on relaxometry uses T1 signal decay instead of T2. T1 values in muscle imaging are known to be increased in case of inflammation and strongly decreased where fat-replacement occurs (89–92); on the contrary, T2 values are elevated in both conditions (93).

Fast T1-mapping sequences have been applied in the study of myocardium (94), whereas only a few studies applied T1 mapping in skeletal muscle imaging in healthy subjects (69, 95), demonstrating a high repeatability.

T1 values, along with T2 and T2^*^ mapping, were found to be significantly increased in a small group of FSHD subjects (*n* = 7) and, in parallel, they correlated well with clinical severity scores (96). Gaur et al. failed to show a T1 abnormality in upper arms in DMD compared to controls (82).

Marty et al. recently explored muscle T1 relaxometry on healthy volunteers and compared its sensitivity with the standard three-points Dixon sequence to assess fat replacement in 30 BMD subjects: the measured T1 values showed a high repeatability, correlating well with FF as measured by Dixon sequence, and could discriminate involved muscles from normality. Additionally the acquisition time was quite fast, with a 10 s scan time per slice (97).

### Diffusivity Imaging

Diffusion imaging reflects the diffusivity of water molecules in biological tissues: in an unrestricted environment, molecules are able to move freely (the so-called free Brownian motion), whereas in biological tissues several structures or conditions, as the presence of cellular membranes, may actually restrict such motion.

In skeletal muscle, water diffusivity increases in active inflammation and decreases where fatty degeneration occurs. As a consequence, diffusion weighted imaging (DWI) can be applied in the assessment of myopathies and may serve as a biomarker for disease course and response to treatment (98, 99).

Diffusion tensor imaging (DTI) is a technical evolution of the concept of diffusion imaging that has been largely applied in brain and spine imaging (100). DTI is based on the ability of water molecules to move differentially in various directions through biologic tissues. The diffusivity along the three axes of the space can be measured applying different gradients; when movement is facilitated in one direction rather than homogeneously in all directions, the so-called anisotropic diffusion occurs, as expressed by fractional anisotropy (FA). Other DTI parameters that assess diffusivity predominantly along the other axes are mean (MD), radial (RD), and axial diffusivity (AD).

The high anisotropic structures found in the human body are the white matter bundles in the central nervous system (CNS) and the muscular fibrillar structures: the well-organized and ordinate muscle proteins and membranous structures represent, in fact, a barrier to free water diffusion, that is conversely allowed along the main axis of the considered structure. When a well-organized structure is disrupted by any physiologic or pathological process, its anisotropy decreases (i.e., FA decreases) (99, 101).

As microstructural abnormalities observed in the context of myopathies, i.e., Z-line abnormalities or increased permeability of cellular membranes, have a negative impact on anisotropic diffusivity, rather facilitating diffusivity along other axes, DTI can play a role to quantitatively investigate such modifications and to monitor the follow-up (99, 102).

When examining DTI data, however, the presence of confounding factors has to be taken into account. A sufficient SNR as well as precise measurements of percentages of fat and mean water T2 are considered essential to reliably estimate the skeletal muscle DTI parameters, limiting confounding effects (103).

The replacement of muscular fibrils by adipose and fibrous tissue in DMD (and by extension in muscular dystrophies in general) actively destroys the barriers that restrict water movement, resulting in an increase in ADC values (apparent diffusivity coefficient, an increased ADC indicates increased free water diffusivity) and a decrease in FA values (decreased integrity of fibrils) (104): ADC and the DTI values can thus be applied to quantitatively assess disease severity in muscle disease (105, 106).

In their pilot study Ponrartana et al. demonstrated a strong correlation between muscle strength and adiposity in boys with DMD, even though such markers could rather represent fat replacement than real muscle damage in later disease stages (105): with regard to diffusivity, muscle strength negatively correlated with FA and positively with ADC. Nonetheless, unexpectedly, more advanced disease correlated positively with FA and negatively with ADC, probably due to artificial increase of ADC and decrease of FA in muscles with >45% of fat replacement.

Another group only observed a trend for the decrease in FA and an increase of mean diffusivity (MD) in the *tibialis anterior* muscle in DMD (103).

DTI analysis has also been used to quantify the muscular diffusivity properties in PM/DM in the field of inflammatory myopathies: ADC and the three eigenvalues (diffusion values in the three axes) were significantly different between normal and affected muscles (99).

Tractography represents an application of DTI that allows the analysis of fibers (in this case of muscle fibers). Muscular architectural aspects such as pennation angle, curvature of fibers, fiber length and possible muscle fibrosis can be studied (107), hence fiber tracking may provide further quantitative information in muscle studies. Intrinsic characteristics of the fibers are influenced by fatty infiltration and muscle atrophy; as a consequence the study of fiber alignment and of diffusivity parameters along the fibers appears of particular interest (102, 108).

Keller et al. demonstrated a strong and consistent association of DTI metrics and fiber total length (FTL) and with mean FF calculated with a Dixon sequence, in a customized ROI segmentation in 15 dystrophic subjects (109). Issues derived from the possibility of incorrect fibers tracking algorithm setting and the effects of partial volume artifacts, however, have to be taken into account (110).

Intra-voxel incoherent motion (IVIM) is another diffusivity based technique that, using several low b values (0–600 s/mm^2^), allows simultaneous quantification of perfusional and diffusivity properties of a tissue (111); with regard to muscle imaging, IVIM has been applied to study perfusion-related muscle damage such as in peripheral arterial occlusive disease and the microstructural muscular properties at rest and following exercise in healthy subjects (112), thus providing a useful alternative to other non-invasive perfusional methods such as arterial spin labeling (ASL, see perfusion section below) (113, 114).

Qi et al. characterized total fluid motion within the muscle in inflammatory myopathies evaluating ADC and the three parameters derived from IVIM, thus separating the diffusion of water molecules from microcapillary perfusion of tissues. In patients, unaffected muscles had DWI coefficients equivalent to those of normal muscles, whereas inflamed muscles showed elevated ADC and diffusion values and decreased volume fraction of capillary perfusion (f); conversely, fat-infiltrated muscles had lower diffusivity values than control muscles, resembling the typical characteristics of fat tissue (98).

Sigmund et al. with a combined Dixon-based and DTI approach applied to thigh and calf muscles in dermatomyositis (DM) observed a lower pseudo-diffusion (D^*^) in the quadriceps and a correlation between diffusion metrics and T1/T2-based scores of disease severity at rest. Additionally, with regard to DTI, post-exercise diffusion measurements showed significantly higher mean (MD) and radial diffusivity (RD) compared to controls (115).

Ran et al. analyzed IVIM in 46 subjects with myopathy and found that standard ADC values along with SNR decay curves and biexponential ADC parameters were useful in distinguishing among PM/DM, glycogen storage diseases, and muscular dystrophy patients (116).

In conclusion, the numerous implementations and possible applications of diffusion weighted imaging can effectively play a relevant role in the management of myopathies, both at rest and after exercise, in the attempt to better describe the underlying pathological processes.

### Magnetization Transfer

Magnetization transfer (MT) is an MRI technique that is able to depict the interactions of tissue-water protons that are part of different macromolecular environments. To simplify using a two-pool picture, water protons in tissues are considered to reside either in the “free” water proton pool (responsible of the conventionally visible MRI signal) or the “restricted” proton pool where protons are bound to proteins and macromolecules. Quantification of MT provides estimates of the relaxation and exchange rates protons between the two pools and the ratio between them (the so called pool size ratio, PSR) (66, 117).

Magnetization transfer contrast is high in skeletal muscle and its origin and mechanisms have been studied by several groups in normal, aging and diseased muscles, with a particular attention for the detection and estimation of fibrosis, even if the link is not completely understood (95, 118–122). In particular, it has been demonstrated that PSR may be used as a biomarker of inflammation, though in a murine model (123).

Sinclair et al. showed that MT ratios (MTR) may be more sensitive to muscle damage than conventional MRI in 10 patients with Charcot Marie Tooth disease type 1A (CMT1A) and nine patients with chronic inflammatory demyelinating polyneuropathy (CIDP) compared to controls. MTR measurement showed correlation with functional measures and with conventional STIR sequences, thus being consistent with visualization of muscular edema; additionally a reduction of MTR in muscles otherwise deemed normal on STIR was found, suggesting an increased sensitivity to ongoing disease (121). Also in CMT1A and sIBM subjects, even after adjustment for FF (that could alter the quantification), MTR was reduced in muscles without substantial intramuscular fat accumulation compared to controls, suggesting sensitivity to early and potentially reversible changes in muscle water distribution (56).

As a further implementation of the technique, MT has been combined with double quantum filtering and ultra-short TE (UTE) imaging, with the aim to improve the technique specificity in detecting collagen, with promising results (124).

### Spectroscopy

Magnetic resonance spectroscopy (MRS) is a technique that is able to provide information regarding the metabolites composing tissues, by separating them according to their unique chemical shifts. The spectroscopic identification of metabolites is achievable applying different techniques; in fact different MR spectroscopies are available, according to the different studied nucleus, ^1^H (also called proton spectroscopy, the more commonly found in practice), ^31^P (phosphorus), ^23^Na (sodium), ^13^C (carbon) being the most important ones (125).

The possibility to detect any abnormality in metabolites that may even precede clinical symptoms and even subtle fat infiltration in skeletal muscle is of particular interest, as it could distinguish early disease-related changes that are potentially reversible and, additionally, track metabolite abnormalities throughout the disease course.

As routine MRI examinations rely on proton signal, the almost totality of clinical scanners are only equipped with the hardware that is necessary to perform proton spectroscopy (^1^H-MRS). Scanning of nuclei other than ^1^H requires dedicated radiofrequency coils to transmit/receive signals and other dedicated hardware and software that are not part of the standard clinical MRI packages.

#### ^31^P MRS

**Figure 4:**
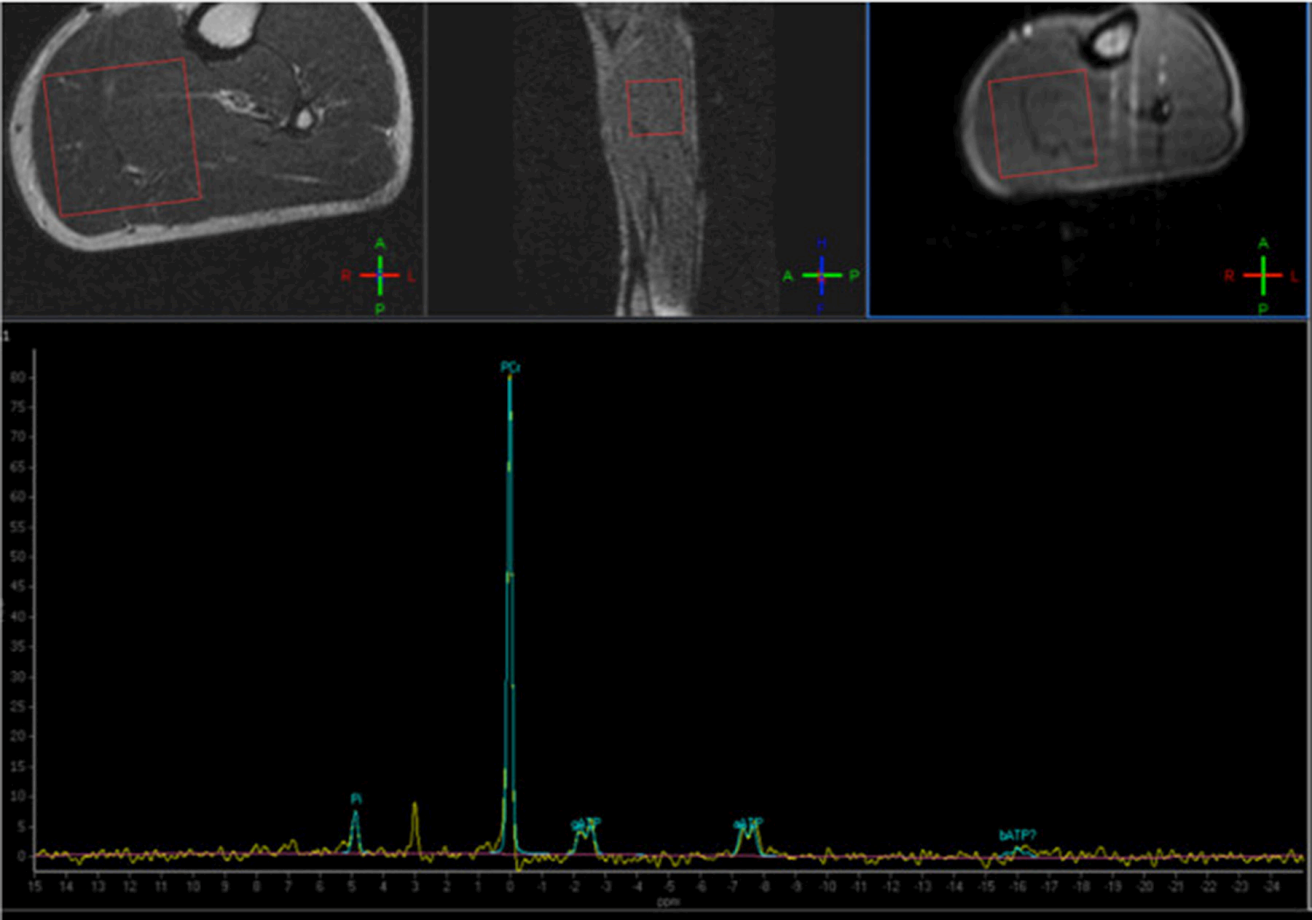
Phosphorus Magnetic Resonance Spectroscopy (^31^P-MRS) of a normal subject, with the voxel positioned at the level of the posterior leg muscles. The normal phosphocreatine peak is evident (Courtesy of Dr. Claudia Cinnante, Neuroradiology Unit, Fondazione IRCCS Ca' Granda Ospedale Maggiore Policlinico, Milan, Italy). Image have been collected during routine patient care.

Despite being the most used and studied in the last decades, the applicability of ^31^P-based MRS in skeletal muscle imaging is nonetheless limited by the need of dedicated equipment, generally not available in a typical clinical setting (Figure 4).

Phosphorus (P) MR spectroscopy has long been used mainly because of its ability to detect signals from biological molecules important in the cellular energetic processes, including phosphocreatine (PCr), adenosine triphosphate (ATP), and inorganic phosphate (Pi) (126–129). Other molecules that are often studied are phosphomonoesters and phosphodiesters (PDE) that are products of degradation of membrane phospholipids (130, 131). Finally, though in an indirect way, also tissue pH can be estimated with this technique (132).

Some studies suggest that P-including metabolites or ratio of metabolites can be used as early disease markers, before significant fat replacement occurs (85, 133). In particular PDE-levels appear as a promising biomarker (134).

In DMD, MR spectroscopy has been applied both to evaluate cellular metabolism and to measure intramuscular lipid composition (85, 135–137). Studies dating back to late eighties and nineties documented decreased phosphocreatine/inorganic phosphate (PCr/Pi) and PCr, but increased Pi in gastrocnemius muscle of DMD children (128, 138). More recently Torriani et al. showed that PCr/Pi of posterior lower leg muscles was significantly lower in DMD children, suggesting that it may be used as a measure of bioenergetic reserve and as a marker of cellular oxidative metabolism (135). They also found elevated muscular pH, in line with previous experiences in literature (128, 138), and suggested that pH increase in DMD may be a response to dystrophin deficiency, possibly related to regenerative mitogenesis or altered ion transport. Importantly, Torriani et al. did not find any correlation between strength and motor functions and spectroscopy parameters, differently from a few small studies conducted previously that went in that direction (nonetheless the small sample sizes of those studies effectively prevented any significant conclusion) (137, 139). As authors suggested, in the context of small sized cohorts, metabolite profiles as defined by MRS may not be as sensitive as MR imaging and measures of fat replacement in predicting disease severity (135).

Hooijmans et al. (134) evaluated calf muscles in DMD subjects with ^31^P MRS at 7 Tesla and found that extensively fat replaced muscles had some metabolite ratios, including PDE/ATP, significantly increased compared to controls. Additionally, only the PDE/ATP ratio (and T2 values, see dedicated section above) was increased in muscles with only a minor fat replacement (85), suggesting PDE/ATP ratio may be used as a biomarker of mild/initial disease. The same group documented that in DMD PDE levels increase 2-fold in muscles with different levels of involvement over 2 years time, supporting the hypothesis that PDE-levels may increase very rapidly early in the disease process and remain elevated thereafter (134).

Phosphorus MRS of the upper limb in patients with DMD has also been attempted, being able to discriminate between patients and controls and also defining a specific pattern of disease evolution in non-ambulant subjects (49, 140).

In BMD Wokke et al. found higher PDE/ATP ratios in several not fat-replaced muscles, whereas PDE/ATP ratios were higher in all measured muscles with increased fat levels compared to healthy controls. Tissue pH was also found increased in all muscles of BMD subjects compared to healthy controls (133). Tosetti et al. found metabolites abnormalities also in mild BMD subjects, similar to previous findings in more severe BMD subjects (128, 141, 142), confirming an increased intracellular pH at rest in BMD (even though the meaning of such a finding is unclear) (143). In particular, the slight increase in the PDE peak could represent an early catabolic marker of disease (144), consistent with other muscular dystrophies (145).

The evaluation of pre- and post-exercise cellular energetics in DMD has not yet been performed; in BMD or DMD/BMD carriers anomalies were shown, but with equivocal results showing bidirectionally abnormal glycolysis, but no real impairment of oxidative phosphorylation (127, 128, 135, 141, 142, 144, 146).

Altered metabolic patterns have also been reported in myotonic dystrophy (147), LGMD (145), and FSHD, generally corresponding to either muscle degeneration or regeneration (148, 149). Furthermore, investigators have shown that ^31^P-containing metabolite concentrations correlated with the extent and rate of fat replacement and strength in FSHD (150).

Finally, also in subjects with inflammatory myopathies, ^31^P MRS showed an impaired phosphorous metabolism during exercise, that tended to normalize after treatment with steroids (90, 151). Other studies, however, have suggested a limited role for such metabolic abnormalities in inflammatory myopathies as sIBM (152).

#### ^1^H MRS (Proton MRS)

Proton (^1^H)-MRS does not require any additional hardware or software to be used in the most widely available MR scanning systems, hence it can easily implemented in clinical studies. Proton spectroscopy is now considered a reference standard for non-invasive fat quantification (153, 154). As fat replacement is a constant pathologic feature of dystrophies and most myopathies, the ability to reliably quantify fat is of primary importance: however, it must be reminded that for a correct application of MRS the water content within the muscle must be consistent throughout, a requisite that is obviously not respected in case of ongoing inflammation/edema, commonly occurring in myopathies (79, 155).

Numerous studies have demonstrated that ^1^H-MRS is a useful method to quantify intramuscular lipid concentrations in dystrophic patients, especially in DMD (135, 137, 156, 157), also showing that MRS profiles may serve as biomarkers in disease progression in longitudinal studies (46). Arpan et al. evaluated with proton MRS the effects of corticosteroids in boys with DMD and demonstrated that MRS profiles can monitor the effects of therapy (48).

In subjects with IIM, proton spectroscopy showed that creatine (Cr) concentrations in normal appearing muscles on T1 and STIR images are increased compared to healthy controls, suggesting that changes in MRS profiles may precede pathological changes on conventional MRI (158).

In conclusion, to date, no pathognomonic proton MR spectroscopy pattern has been found to identify a specific myopathy, as fatty degeneration is the last step of them all (132). In such a context, spectroscopy of other nuclei may rather be a useful tool in the assessment of damage even before fat replacement and/or functional impairment occur, and may also track treatment response.

#### ^23^Na MRS

Sodium (Na) plays a vital role in cellular function and integrity; it is found both in the intracellular and extracellular space, but its extracellular concentration is 8–10 times higher than the intracellular one, the sodium-potassium (Na^+^-P^+^) pump being the responsible agent for the maintenance of the concentration gradient. In myocites the abnormalities in total sodium concentration and in its trans-membrane flux rates occur in several physiological and pathological conditions, including exercise, cellular proliferation, and myopathies (159–162).

As sodium is less represented in tissues than hydrogen, Na MRS has an average SNR that is extremely lower than that of proton MRS (additionally depending on organs). Such issue has been at least partially resolved by the progressive implementation of higher magnetic field scanners, up to 9.4 T (163), which increase the SNR.

With regard to DMD, the sodium concentration has been shown to be elevated in the myoplasm, with an osmotic effect causing intracellular muscle edema (164). In a pilot study, Glemser et al. investigated the effects of eplerenone in two DMD subjects and found that intracellular-weighted sodium signal and muscular sodium concentrations decreased with therapy (along with edema), suggesting a role of sodium quantification by MRS as a tracer of disease activity/response to therapy (165).

Myotonic dystrophy has also been linked to sodium channel conductance dysregulation, which may pathologically increase muscle fiber concentrations, influencing disease severity (161); in addition to this, the dysregulation was even incremented after exercise (162).

As aforementioned, the development of sodium spectroscopy at higher magnetic field strengths of 3T or 7T allows a more elevated SNR and thus a more precise quantification of intracellular ^23^Na homeostasis in healthy volunteers and in myopathies. The potential of ^23^Na MRI at ultra-high fields can be expected to be promising in the near future.

#### ^13^C MRS

No studies have used carbon spectroscopy yet to examine either inherited or acquired myopathies. Nonetheless, this technique has been used to investigate skeletal muscle metabolism, mainly to determine glycogen levels in normal subjects and in altered glycogen storage diseases (166–170).

As already seen for sodium and phosphorus, carbon imaging is, however, quite challenging, as it requires dedicated equipment uncommonly found in a clinical setting.

#### Multi-Voxel MRS

Multi-voxel MRS is a technical advancement in spectroscopic studies, largely applied in the neuroradiological domain, which may possibly play a role in muscular imaging, given its ability to simultaneously image multiple voxels in a single acquisition. Traditional single-voxel spectroscopy, in fact, suffers the same issues as a routine muscular biopsy, as it focuses on a chosen region of interest, whereas the disease could involve an entire muscle or different parts of it in a heterogeneous manner. In this sense, the single-voxel approach could miss areas of disease pathology, showing false negative results.

Previously multiple-voxel MRS required a number of technical steps that significantly increased scan time, thus greatly limiting its applications: the recent implementations of various fast MRS techniques (as multiple-echo acquisition, echo-planar spectroscopy imaging, and parallel encoded MRS), however, has fortunately reduced the total scan time, making the multi-voxel approach more attractive, also in a clinical setting. As for the central nervous system, the obtained metabolites spectra can then be plotted and superimposed to anatomical images of muscles for a better overall depiction of the real involvement of rather large anatomic regions.

### MRI Elastography (MRE)

Magnetic resonance elastography (MRE) assesses the mechanical properties of soft tissues by analyzing the propagation of mechanical waves in the tissue, being considered as an imaging-based counterpart to palpation. The magnetic resonance signal can be sensitized to motion down to a submillimetric level and can effectively track the propagation in tissues of such pressure waves. The velocity of the propagation is directly proportional to tissue stiffness: as a consequence, it can be used to estimate the viscoelastic properties of tissues, stiffness above all (171, 172). More in detail, MRE functions as follows: an external mechanical device, whose function is regulated by the MR pulse sequence, generates the shear waves, which propagate through the muscular tissue. MR detection of such wave propagation results in the quantification of tissue elasticity (i.e., stiffness), through the generation of elasticity parameters maps, called elastograms. One of the main limitations of MRE approach is, however, linked to the manual nature of the stimulus, which can suffer from variations (173).

MRE can be used to assess muscular physiologic responses and, more often, stiffness in normal and damaged muscles (174–177).

In a MRE study in DMD, muscular stiffness was increased in upper and lower limb muscles compared to healthy muscles, the greatest increases being in the most proximal groups (178). Conversely, in myositis, muscle stiffness of involved muscles was decreased at MRE, supporting the presence of an inflammatory injury to the muscular tissue (179).

Tissue elastography can also be evaluated using ultrasound (US) methods, with results comparable to MRE and with a clear advantage in terms of feasibility, patient acceptance and general availability of the technical equipment (180, 181). The feasibility and reliability of US elastography has not only been validated for strain elastography but also for shear-wave elastography (SWE) (180, 182).

Dastgir et al. evaluated SWE values in a large mixed cohort of subjects with myopathy (*n* = 45), finding only three muscles with values different from healthy controls (183, 184). Also Zaidman et al. confirmed that US quantitative measures can effectively detect abnormalities in dystrophic muscles, by using a different US quantitative measure called calibrated muscle backscatter ultrasound echointensity (185).

Pichiecchio et al. evaluated five DMD preschool children with a combined approach including conventional MRI (via a semi-quantitative analysis) and shear-wave elastography, suggesting that SWE could be considered a more sensitive instrument than MRI for detecting early muscle changes (186). If confirmed in larger cohorts, such data could open interesting scenarios for SWE application in myopathies.

### Other Techniques of Advanced Imaging

#### Perfusion Imaging

Even if functional MRI (fMRI) is immediately associated to functional studies of the brain, this technique has also been applied to other organs, including skeletal muscles, to assess microvasculature (187–189). The BOLD (blood oxygen level dependent) contrast used to build the fMRI signal, in effect, is based on alterations in hemoglobin oxygenation: the paramagnetic deoxyhemoglobin, in fact, distorts the local magnetic field, consequently influencing the local signal.

The skeletal muscle BOLD signal reflects the muscular microcirculation and can be regarded as a non-invasive tool to indirectly evaluate muscular function (188, 190, 191). BOLD fMRI has been applied to examine diseases causing altered perfusion of skeletal muscles (192, 193) and to assess the brain response to motor stimulation in myotonic dystrophy, but not at muscular level (194). Despite the known muscle degeneration detected in dystrophic patients, no altered microcirculation has been found in these patients so far.

Arterial spin labeling (ASL) is an advanced MR technique for measuring local perfusion without administration of intravenous gadolinium, mostly applied in brain imaging, particular in a pediatric setting. Skeletal muscle ASL has been validated several years ago (195), but a few more recent ASL studies always of normal muscles are available even at higher magnetic field up to 7 Tesla (196–198). Reports of clinical application of ASL in a pathologic context are scarce, including compartment syndrome and peripheral artery disease (199–201). Important limitations include known sensitivity of ASL technique to motion artifacts, low contrast-to-noise ratio, and relatively long acquisition times (202).

#### Contrast Enhanced Imaging

Contrast administration for MR muscle imaging is an off-label practice in clinical context of myopathies and its availability and applicability vary by jurisdiction and over time (203).

Gadolinium has been thoroughly used as a marker of fibrosis in cardiac imaging, where the replacement of myocardial cells is associated with the expansion of the interstitial space (204). Delayed contrast enhancement in cardiac imaging is a powerful marker to identify myocardial replacement by connective tissue. Yet a prerequisite for this approach is the absence of extracellular edema or any situation that could cause pathological enhancement of myocardial cells, such as necrosis or inflammation. In such cases, delayed enhancement is not reliable in documenting fibrosis.

Gadolinium enhancement could be applied to indirectly evaluate fibrosis in the skeletal muscle as well, but no studies are currently available in literature, probably due to several technical issues including the smaller extracellular space and lesser degree of fibrosis characteristic of skeletal muscle (29).

#### Dynamic MRI

The applicability of electrical muscular stimulators (EMS) that are MRI-compatible has been recently demonstrated (205, 206). As a consequence, interest has risen regarding the possibility to study the movement-linked micro-changes in muscular tissue, using MR sequences that are synchronized with the electrical stimuli. Deligianni et al. demonstrated that both qualitative and quantitative parameters that have a significant dependence on stimulation levels (i.e., stimulation current) can be extracted from the integration of EMS-based stimulus and MRI (206). A wide range of triggered MRI sequences, including conventional cardiovascular sequences, can be used in combination with this technique; the possible scenarios of application of stimulus MRI are quite broad.

#### Texture Analysis

The human eye is capable to detect the visual patterns such as roughness and smoothness of an image, and specifically in diagnostic images, although with physiological limits that prevent the detection of subtle variations, such as those of thousands of gray levels, which is something a computer can effectively do.

Texture analysis is a computerized method that can detect the signal from each pixel of an image and describe the spatial variations of pixel signals, allowing a deeper insight into the image structure that may be impossible to detect visually; this way, texture analysis can indeed reflect in detail the microstructural heterogeneity of signal with a good sensitivity, in a non-operator-dependent way. This approach has been applied in several imaging modalities, to precisely characterize even the smallest variations of tissue homogeneity (207).

In 1999, Herlidou et al. demonstrated that texture analysis could discriminate between healthy subjects and dystrophic patients muscles with a sensitivity of 70%, and a specificity of 86% (208). Arpan et al. built T2 signal histograms of lower leg muscles in DMD and showed that they can represent an alternative approach to quantify disease involvement. A threshold was applied to quantify the percentage of affected areas: when compared to conventional method using mean T2 muscle signal, the supra-threshold pixels could better monitor involvement in dystrophic muscles. Therefore, the authors suggested that such a method may be more sensitive in the follow-up of minor pathologic linked to the disease evolution (77). For instance, T2w- and contrast-enhanced T1w-based histogram analysis has been implemented recently to assess the radiation changes in internal obturator muscles after radiotherapy for prostate cancer (209) and in paravertebral muscle of symptomatic lumbar canal stenosis (210).

Some studies applied histogram analysis to muscle ultrasonographic images, using different measures of tissue homogeneity, in healthy subjects (211, 212); others applied it to muscle disorders and found that disease involvement (here DMD) could effectively be well-described with such a technique and with a high level of detail (213). In a more recent study of muscle US, Sogawa et al. showed that texture analysis of ultrasonographic data can differentiate between neurogenic and myogenic diseases being promisingly useful in the non-invasive assessing of the underlying disease mechanisms (214).

Several limitations, however, apply. First, this analytic approach is computationally demanding and needs dedicated software. As already underlined by Carlier et al. texture analysis additionally depends on the used voxel size. A large voxel would, in fact, delete subtle differences and make the image too homogeneous with consequent loss of information, a small voxel would be largely influenced by rumor with possible confounding effect on the texture (29). Furthermore, the whole analyzed region has to be chosen above a certain size, to limit the effect of partial volume (209, 215). A harmonization of acquisition parameters between centers who apply such a technique is thus required; if correctly implemented and regulated, however, image texture analysis of MR or US images can be regarded as a promising diagnostic tool (216, 217).

#### Nuclear Medicine

In addition to its central importance in oncological imaging, positron emission tomography (PET) has nevertheless gained an increasing role in the detection of muscle inflammation, also in the clinical setting. A flogistic process of whatsoever origin is, in fact, associated with increased uptake of ^18^F-FDG. Yet, even if PET sensitivity in the detection of high-grade inflammation (e.g., acute infection) is high, the sensitivity in the context of inflammatory myopathies is relatively low. In such cases the combination of PET and MR imaging (i.e., T2-weighted sequences to detect edema) are recommended to increase the accuracy of diagnosis (218–220).

To date no study including a large cohort has been performed to highlight the value of ^18^F-FDG PET in the setting of non-inflammatory myopathies, the main reason being the high availability of conventional US and/or MRI imaging and the general tendency of physicians to avoid radiation exposure in non-oncologic subjects.

Liau et al. presented a case of a man with dermatomyositis in which PET/CT imaging revealed diffuse proximal muscle hypermetabolism, consistent with myositis inflammation (221). Renard et al. reported a case of DM in which the initial FDG-PET showed diffuse, increased, proximal predominant, muscular uptake, later normalized after steroid therapy (222). Others investigated the value of PET in different types of inflammatory myopathies, yet only in four cases: two subjects with low-grade myositis and one cases of sIBM were negative at PET examinations, whereas a case of DM showed diffuse proximal muscle hypermetabolism (223): the authors comment that FDG-PET is able to detect more severe myopathies when necrotizing/inflammatory changes occur, helping in demonstrating response to treatment. Nevertheless, as aforementioned, the role of PET in low-grade disease needs further assessment, due to reported low sensitivity in mild cases.

In 20 subjects, Tanaka et al. showed that FDG uptake can discriminate PM/DM from non-muscular diseases and is highly sensitive in detecting muscle inflammation in proximal muscles: moreover, the FDG uptake correlated well with muscle weakness and with the presence of inflammatory cells at biopsy (224). Other authors instead underlined how visual assessment of FDG uptake and the quantification of standardized uptake value (SUV) can have a significant role in the clinical management of PM/DM, allowing a better understanding of the diseases (225). In a larger cohort of 38 IIMs, Li et al. found that muscular FDG-PET uptake was generally higher in patients than controls, and that muscular uptake correlated with muscle strength and serum creatine kinase (CK) levels. Furthermore, PET examination, whose primary purpose was to rule out any malignancy in the context of IIMs, succeeded in detecting a tumor in roughly 20% of cases (226).

Also an interesting, large-cohort, single-photon emission computed tomography (SPECT) study applying ^99m^Tc-PYP (technetium pyrophosphate) in 90 IIMs subjects confirmed that radiotracer uptake was significantly higher than in healthy controls (227).

As per brain imaging, other PET tracers such as Pittsburg compound B (PIB) may also play a role in muscle imaging. A few experiences in the detection of amyloid plaques by PIB-PET in myocardium or in skeletal muscles in sIBM were remarkable (228, 229).

Finally, the recent implementation and increasing diffusion of new PET/MR scanners as the hybrid imaging technique of choice beyond the traditional PET/CT imaging, will probably improve the imaging process and promote an advance in knowledge also in the context of myopathies and muscle diseases in general (230).

#### Whole-Body MRI (WB-MRI)

Muscle MR scans are usually focused on certain ROIs for a number of reasons (firstly due to time constraints limiting too long acquisitions) based on the clinical suspicions. The technical implementation of whole-body MRI (WB-MRI) has overcome such “limitation,” making it possible to contemporarily study the entire body in a reasonable time (typically 1 h or less) (231, 232), assessing at a time fat and muscular volume (23, 233–236).

WB-MRI can effectively provide information that is enormously useful to clinical evaluation, detecting all areas of inflammatory involvement or fat replacement throughout the body, greatly helping in the diagnostic process (237). It consequently allows a better tailoring of eventual treatments, and, last but not least, a better monitoring of their efficacy, in a reasonably short time. WB-MRI can also serve as guidance for muscular biopsy possibly even more than conventional MRI, given the wide field of view, in particular for uncertain cases.

**Figure 5:**
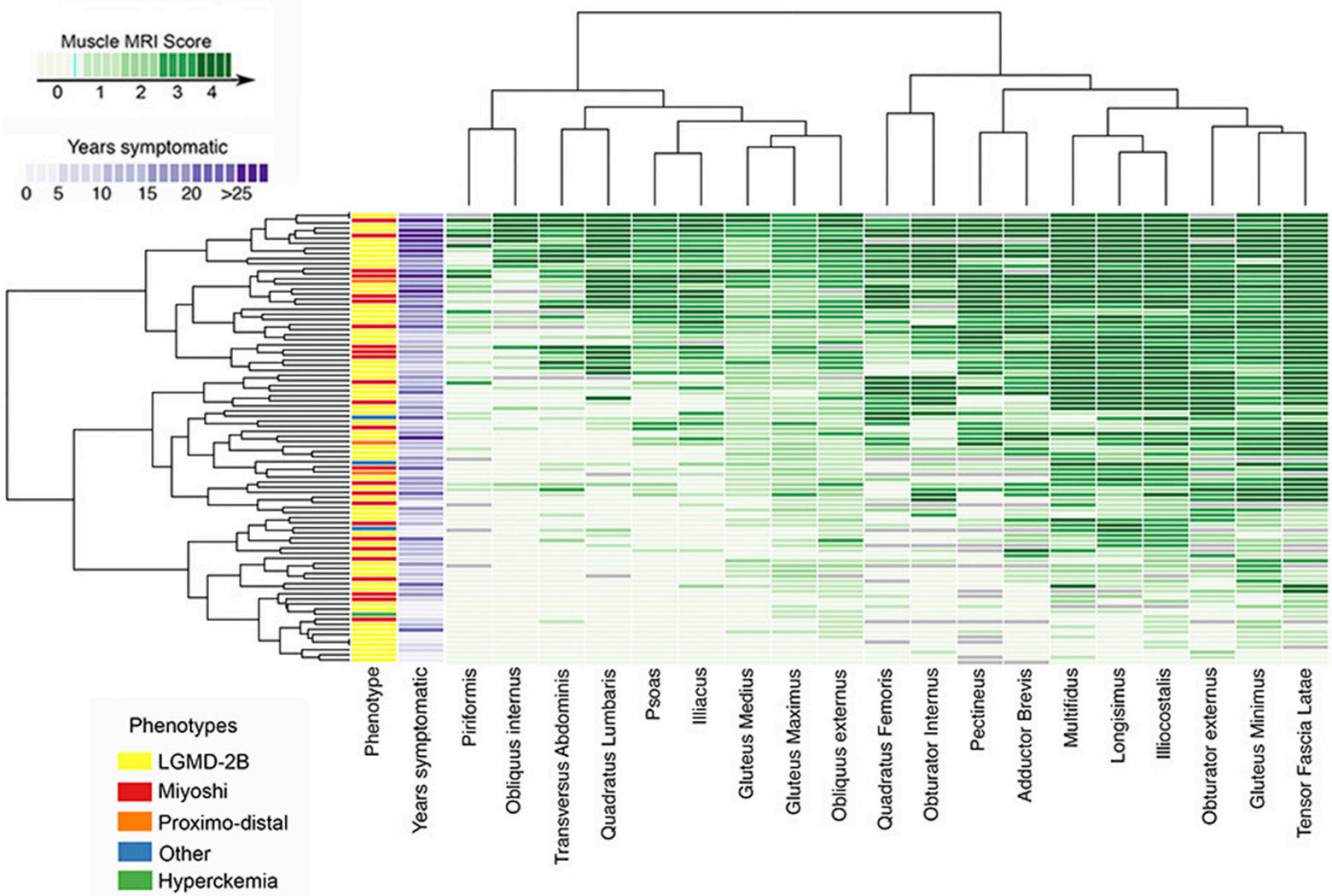
Heatmap of pelvic muscle involvement in disferlynopathies. The map displays the Mercuri score for all pelvic muscles in 182 subjects, also reporting the different phenotypes and the number of years in which the patient was symptomatic. Subjects are arranged (left to right) from lowest to highest mean MRI score (Courtesy of Dr. J. Diaz Manera, Neurology Service, Hospital de la Santa Creu i Sant Pau, Barcelona, Spain). The presented heatmap is a visual representation and exemplification of the Mercuri score: the MRI conventional images used for building the heatmap have been collected for routine patient care.

Conversely, the analysis of WB-MRI datasets may represent a difficulty, given the large amount of acquired data. To address such an issue, Hankiewicz et al. have proposed the use of a graphical technique (so called heat maps), already applied in other scientific disciplines, which provides a visual representation of each muscle of the body (Figure 5). Hence, a visualization of the overall condition of muscles of a subject at a glance is possible (23) and involved and/or spared muscles become immediately evident at visual inspection (23, 238). Such a reader-friendly visual presentation of data, in fact, could make more evident or reveal subtle relationships, tendencies, and involvement patterns (238), building a sort of a “fingerprint” of a subject, and, as a further step, of a specific disease. The so-built heat maps can thus be applied to promptly recognize a myopathic pattern and to facilitate uncertain diagnosis (236, 238, 239).

To date applied protocols for WB-MRI generally include traditional sequences as T1, T2 and STIR; nonetheless an effort is made to extend studies including DWI and other advanced techniques, with promising scenarios.

## Conclusions

The recent advances in imaging techniques have greatly changed the diagnostic approach in the field of neuromuscular diseases. Although clinical evaluation remains the pivot of the diagnostic process and muscle biopsy is often an essential procedure in order to correctly define the diagnosis, there is no doubt that current imaging techniques have taken a prominent place in the muscle disease work-up.

The application in clinical practice of advanced quantitative techniques will certainly lead to a further evolution in the approach to this group of diseases. Thereby, the diagnostic process becomes increasingly a “multidisciplinary affair” involving the clinical myologist, the myopathologist, the geneticist, and the neuroradiologist. A proper amalgam of all these skills is undoubtedly essential and represents the future of myology.

## Author Contributions

MP and AP: acquisition of data, analysis and interpretation of data, drafting of manuscript. SC: drafting of manuscript. GT, AB, and AlP: critical revision. MF: study conception and design, drafting of manuscript, critical revision.

### Conflict of Interest Statement

The authors declare that the research was conducted in the absence of any commercial or financial relationships that could be construed as a potential conflict of interest.

## Abbreviations

ASL: arterial spin labeling
MBD: Becker muscular dystrophy
BOLD: blood oxygenation level dependent
DMD: Duchenne muscular dystrophy
BMD: Becker muscular dystrophy
DWI: diffusion weighted imaging
DTI: diffusion tensor imaging
FF: fat fraction
FSHD: facio-scapulo-humeral muscular dystrophy
IBM: inclusion body myositis
IDEAL: iterative decomposition of water and fat with echo asymmetry and least-squares estimation
LGMD: limb girdle muscular dystrophy
LOPD: late onset Pompe disease
MD: myotonic dystrophy
MR: magnetic resonance
MRE: magnetic resonance elastography
MRI: magnetic resonance imaging
MRS: magnetic resonance spectroscopy
MT: magnetization transfer
OPMD: oculopharingeal muscular dystrophy
PET: positron emission tomography
qMRI: quantitative magnetic resonance imaging
SEP1N: selenoprotein N
SNR: signal to noise ratio
STIR: short-tau inversion recovery
SWE: shear wave elastography
TSE: turbo spin echo
US: ultrasound.

## References

[1] Domingo-Horne RM Salajegheh MK. An approach to myopathy for the primary care clinician. Am J Med. (2018) 131:237–43. 10.1016/j.amjmed.2017.10.016 29074094

[2] Savarese M Di Fruscio G Torella A Fiorillo C Magri F Fanin M. The genetic basis of undiagnosed muscular dystrophies and myopathies: results from 504 patients. Neurology (2016) 87:71–6. 10.1212/WNL.0000000000002800 27281536 PMC4932234

[3] Mercuri E Pichiecchio A Counsell S Allsop J Cini C Jungbluth H. A short protocol for muscle MRI in children with muscular dystrophies. Eur J Paediatr Neurol. (2002) 6:305–7. 10.1053/ejpn.2002.0617 12401454

[4] Fischer D Kley RA Strach K Meyer C Sommer T Eger K. Distinct muscle imaging patterns in myofibrillar myopathies. Neurology (2008) 71:758–65. 10.1212/01.wnl.0000324927.28817.9b 18765652 PMC2583436

[5] Costa AF Di Primio GA Schweitzer ME. Magnetic resonance imaging of muscle disease: a pattern-based approach. Muscle Nerve (2012) 46:465–81. 10.1002/mus.23370 22987686

[6] Maurer B Walker UA. Role of MRI in diagnosis and management of idiopathic inflammatory myopathies. Curr Rheumatol Rep. (2015) 17:67. 10.1007/s11926-015-0544-x 26385754

[7] Pipitone N Notarnicola A Levrini G Spaggiari L Scardapane A Iannone F. Do dermatomyositis and polymyositis affect similar thigh muscles? A comparative MRI-based study. Clin Exp Rheumatol. (2016) 34:1098–100. 27991408

[8] de Castro Miranda SS Alvarenga D Rodrigues JC Shinjo SK Different aspects of magnetic resonance imaging of muscles between dermatomyositis and polymyositis. Rev Bras Reumatol. (2014) 54:295–300. 10.1016/j.rbr.2014.04.004 25627225

[9] Davis WR Halls JE Offiah AC Pilkington C Owens CM Rosendahl K. Assessment of active inflammation in juvenile dermatomyositis: a novel magnetic resonance imaging-based scoring system. Rheumatology (2011) 50:2237–44. 10.1093/rheumatology/ker262 21972421

[10] Dion E Cherin P Payan C Fournet JC Papo T Maisonobe T. Magnetic resonance imaging criteria for distinguishing between inclusion body myositis and polymyositis. J Rheumatol. (2002) 29:1897–906. 12233884

[11] Polavarapu K Manjunath M Preethish-Kumar V Sekar D Vengalil S Thomas PT. Muscle MRI in Duchenne muscular dystrophy: evidence of a distinctive pattern. Neuromusc Disord. (2016) 26:768–74. 10.1016/j.nmd.2016.09.002 27666775

[12] Barp A Bello L Caumo L Campadello P Semplicini C Lazzarotto A. Muscle MRI and functional outcome measures in Becker muscular dystrophy. Sci Rep. (2017) 7:16060. 10.1038/s41598-017-16170-2 29167533 PMC5700122

[13] Mercuri E Bushby K Ricci E Birchall D Pane M Kinali M. Muscle MRI findings in patients with limb girdle muscular dystrophy with calpain 3 deficiency (LGMD2A) and early contractures. Neuromusc Disord. (2005) 15:164–71. 10.1016/j.nmd.2004.10.008 15694138

[14] Fischer D Walter MC Kesper K Petersen JA Aurino S Nigro V. Diagnostic value of muscle MRI in differentiating LGMD2I from other LGMDs. J Neurol. (2005) 252:538–47. 10.1007/s00415-005-0684-4 15726252

[15] Illa I De Luna N Domínguez-Perles R Rojas-García R Paradas C Palmer J. Symptomatic dysferlin gene mutation carriers: characterization of two cases. Neurology (2007) 68:1284–9. 10.1212/01.wnl.0000256768.79353.60 17287450

[16] Diaz-Manera J Fernandez-Torron R LLauger J James MK Mayhew A Smith FE. Muscle MRI in patients with dysferlinopathy: pattern recognition and implications for clinical trials. J Neurol Neurosurg Psychiatry (2018) 89:1071–81. 10.1136/jnnp-2017-317488 29735511 PMC6166612

[17] Tasca G Monforte M Díaz-Manera J Brisca G Semplicini C D'Amico A. MRI in sarcoglycanopathies: a large international cohort study. J Neurol Neurosurg Psychiatry (2018) 89:72–7. 10.1136/jnnp-2017-316736 28889091

[18] Sandell SM Mahjneh I Palmio J Tasca G Ricci E Udd BA. “Pathognomonic” muscle imaging findings in DNAJB6 mutated LGMD1D. Eur J Neurol. (2013) 20:1553–9. 10.1111/ene.12239 23865856

[19] Tasca G Monforte M Ottaviani P Pelliccioni M Frusciante R Laschena F. Magnetic resonance imaging in a large cohort of facioscapulohumeral muscular dystrophy patients: pattern refinement and implications for clinical trials. Ann Neurol. (2016) 79:854–64. 10.1002/ana.24640 26994363

[20] Wattjes MP Kley RA Fischer D. Neuromuscular imaging in inherited muscle diseases. Eur Radiol. (2010) 20:2447–60. 10.1007/s00330-010-1799-2 20422195 PMC2940021

[21] Jungbluth H Davis MR Müller C Counsell S Allsop J Chattopadhyay A. Magnetic resonance imaging of muscle in congenital myopathies associated with RYR1 mutations. Neuromusc Disord. (2004) 14:785–90. 10.1016/j.nmd.2004.08.006 15564033

[22] Mercuri E Clements E Offiah A Pichiecchio A Vasco G Bianco F. Muscle magnetic resonance imaging involvement in muscular dystrophies with rigidity of the spine. Ann Neurol. (2010) 67:201–8. 10.1002/ana.21846 20225280

[23] Hankiewicz K Carlier RY Lazaro L Linzoain J Barnerias C Gómez-Andrés D. Whole-body muscle magnetic resonance imaging in SEPN1-related myopathy shows a homogeneous and recognizable pattern. Muscle Nerve (2015) 52:728–35. 10.1002/mus.24634 25808192

[24] Alejaldre A Díaz-Manera J Ravaglia S Tibaldi EC D'Amore F Morís G. Trunk muscle involvement in late-onset Pompe disease: study of thirty patients. Neuromusc Disord. (2012) 22 (Suppl. 2):S148–54. 10.1016/j.nmd.2012.05.011 22980766

[25] Pichiecchio A Uggetti C Ravaglia S Egitto MG Rossi M Sandrini G. Muscle MRI in adult-onset acid maltase deficiency. Neuromusc Disord. (2004) 14:51–5. 10.1016/j.nmd.2003.08.003 14659413

[26] Carlier RY Laforet P Wary C Mompoint D Laloui K Pellegrini N. Whole-body muscle MRI in 20 patients suffering from late onset Pompe disease: involvement patterns. Neuromusc Disord. (2011) 21:791–9. 10.1016/j.nmd.2011.06.748 21803581

[27] Kumbhare DA Elzibak AH Akbari A Noseworthy MD Advanced skeletal muscle MR imaging approaches in the assessment of muscular dystrophies. Int J Phys Med Rehabil. (2014) 2:248 10.4172/2329-9096.1000248

[28] Kalia V Leung DG Sneag DB Grande F Carrino JA Advanced MRI techniques for muscle imaging. Semin Musculoskelet Radiol. (2017) 21:459–69. 10.1055/s-0037-1604007 28772322 PMC5749928

[29] Carlier PG Marty B Scheidegger O Loureiro De Sousa P Baudin PY Snezhko E. Skeletal muscle quantitative nuclear magnetic resonance imaging and spectroscopy as an outcome measure for clinical trials. J Neuromusc Dis. (2016) 3:1–28. 10.3233/JND-160145 27854210 PMC5271435

[30] Kim HK Lindquist DM Serai SD Mariappan YK Wang LL Merrow AC. Magnetic resonance imaging of pediatric muscular disorders. Recent advances and clinical applications. Radiol Clin North Am. (2013) 51:721–42. 10.1016/j.rcl.2013.03.002 23830795 PMC3950969

[31] Burakiewicz J Sinclair CDJ Fischer D Walter GA Kan HE Hollingsworth KG. Quantifying fat replacement of muscle by quantitative MRI in muscular dystrophy. J Neurol. (2017) 264:2053–67. 10.1007/s00415-017-8547-3 28669118 PMC5617883

[32] Ma J. Dixon techniques for water and fat imaging. J Magn Reson Imaging (2008) 28:543–58. 10.1002/jmri.21492 18777528

[33] Del Grande F Santini F Herzka DA Aro MR Dean CW Gold GE Fat-suppression techniques for 3-T MR imaging of the musculoskeletal system. Radiographics (2014) 34:217–33. 10.1148/rg.341135130 24428292 PMC4359893

[34] Mito S Ishizaka K Nakanishi M Sugimori H Hamaguchi H Tsuzuki T. [Comparison of fat suppression techniques of bilateral breast dynamic sequence at 3.0 T: utility of three-point DIXON technique] 2391. Nihon Hoshasen Gijutsu Gakkai Zasshi (2011) 67:654–60. 10.6009/jjrt.67.654 21720074

[35] Barger AV DeLone DR Bernstein MA Welker KM. Fat signal suppression in head and neck imaging using fast spin-echo-IDEAL technique. Am J Neuroradiol. (2006) 27:1292–4. 16775282 PMC8133933

[36] Triplett WT Baligand C Forbes SC Willcocks RJ Lott DJ DeVos S. Chemical shift-based MRI to measure fat fractions in dystrophic skeletal muscle. Magn Reson Med. (2014) 72:8–19. 10.1002/mrm.24917 24006208 PMC4307808

[37] Wren TAL Bluml S Tseng-Ong L Gilsanz V. Three-point technique of fat quantification of muscle tissue as a marker of disease progression in Duchenne muscular dystrophy: preliminary study. AJR Am J Roentgenol. (2008) 190:W8–12. 10.2214/AJR.07.2732 18094282

[38] Hu HH Kan HE. Quantitative proton MR techniques for measuring fat. NMR Biomed. (2013) 26:1609–29. 10.1002/nbm.3025 24123229 PMC4001818

[39] Hooijmans MT Niks EH Burakiewicz J Anastasopoulos C van den Berg SI van Zwet E. Non-uniform muscle fat replacement along the proximodistal axis in Duchenne muscular dystrophy. Neuromusc Disord. (2017) 27:458–64. 10.1016/j.nmd.2017.02.009 28302391

[40] Hollingsworth KG de Sousa PL Straub V Carlier PG. Towards harmonization of protocols for MRI outcome measures in skeletal muscle studies: consensus recommendations from two TREAT-NMD NMR workshops, 2 May 2010, Stockholm, Sweden, 1-2 October 2009, Paris, France. Neuromusc Disord. (2012) 22(Suppl. 2):S54–67. 10.1016/j.nmd.2012.06.005 22980769

[41] Wokke BH Bos C Reijnierse M Van Rijswijk CS Eggers H Webb A. Comparison of dixon and T1-weighted MR methods to assess the degree of fat infiltration in duchenne muscular dystrophy patients. J Magn Reson Imaging (2013) 38:619–24. 10.1002/jmri.23998 23292884

[42] Lamminen AE. Magnetic resonance imaging of primary skeletal muscle diseases: patterns of distribution and severity of involvement. Br J Radiol. (1990) 63:946–50. 10.1259/0007-1285-63-756-946 2268764

[43] Kinali M Arechavala-Gomeza V Cirak S Glover A Guglieri M Feng L. Muscle histology vs MRI in Duchenne muscular dystrophy. Neurology (2011) 76:346–53. 10.1212/WNL.0b013e318208811f 21263136 PMC3034418

[44] Wokke BH van den Bergen JC Versluis MJ Niks EH Milles J Webb AG. Quantitative MRI and strength measurements in the assessment of muscle quality in Duchenne muscular dystrophy. Neuromusc Disord. (2014) 24:409–16. 10.1016/j.nmd.2014.01.015 24613733

[45] Ricotti V Evans MRB Sinclair CDJ Butler JW Ridout DA Hogrel JY. Upper limb evaluation in duchenne muscular dystrophy: fat-water quantification by MRI, muscle force and function define endpoints for clinical trials. PLoS ONE (2016) 11:e0162542. 10.1371/journal.pone.0162542 27649492 PMC5029878

[46] Willcocks RJ Rooney WD Triplett WT Forbes SC Lott DJ Senesac CR. Multicenter prospective longitudinal study of magnetic resonance biomarkers in a large duchenne muscular dystrophy cohort. Ann Neurol. (2016) 79:535–47. 10.1002/ana.24599 26891991 PMC4955760

[47] Willcocks RJ Triplett WT Forbes SC Arora H Senesac CR Lott DJ. Magnetic resonance imaging of the proximal upper extremity musculature in boys with Duchenne muscular dystrophy. J Neurol. (2017) 264:64–71. 10.1007/s00415-016-8311-0 27778157 PMC5226881

[48] Arpan I Willcocks RJ Forbes SC Finkel RS Lott DJ Rooney WD. Examination of effects of corticosteroids on skeletal muscles of boys with DMD using MRI and MRS. Neurology (2014) 83:974–80. 10.1212/WNL.0000000000000775 25098537 PMC4162304

[49] Hogrel JY Wary C Moraux A Azzabou N Decostre V Ollivier G. Longitudinal functional and NMR assessment of upper limbs in Duchenne muscular dystrophy. Neurology (2016) 86:1022–30. 10.1212/WNL.0000000000002464 26888987 PMC4799716

[50] Fischmann A Hafner P Gloor M Schmid M Klein A Pohlman U. Quantitative MRI and loss of free ambulation in Duchenne muscular dystrophy. J Neurol. (2013) 260:969–74. 10.1007/s00415-012-6733-x 23138982

[51] Janssen B Voet N Geurts A Van Engelen B Heerschap A. Quantitative MRI reveals decelerated fatty infiltration in muscles of active FSHD patients. Neurology (2016) 86:1700–7. 10.1212/WNL.0000000000002640 27037227

[52] Bonati U Schmid M Hafner P Haas T Bieri O Gloor M. Longitudinal 2-point dixon muscle magnetic resonance imaging in becker muscular dystrophy. Muscle Nerve (2015) 51:918–21. 10.1002/mus.24629 25736228

[53] Fischer D Hafner P Rubino D Schmid M Neuhaus C Jung H. The 6-minute walk test, motor function measure and quantitative thigh muscle MRI in Becker muscular dystrophy: a cross-sectional study. Neuromusc Disord. (2016) 26:414–22. 10.1016/j.nmd.2016.04.009 27209345

[54] Fischmann A Hafner P Fasler S Gloor M Bieri O Studler U. Quantitative MRI can detect subclinical disease progression in muscular dystrophy. J Neurol. (2012) 259:1648–54. 10.1007/s00415-011-6393-2 22297459

[55] Willis TA Hollingsworth KG Coombs A Sveen ML Andersen S Stojkovic T. Quantitative muscle MRI as an assessment tool for monitoring disease progression in LGMD2I: a multicentre longitudinal study. PLoS ONE (2013) 8:e70993. 10.1371/journal.pone.0070993 23967145 PMC3743890

[56] Morrow JM Sinclair CDJ Fischmann A Machado PM Reilly MM Yousry TA. MRI biomarker assessment of neuromuscular disease progression: a prospective observational cohort study. Lancet Neurol. (2016) 15:65–77. 10.1016/S1474-4422(15)00242-2 26549782 PMC4672173

[57] Gaeta M Mileto A Mazzeo A Minutoli F Di Leo R Settineri N. MRI findings, patterns of disease distribution, and muscle fat fraction calculation in five patients with Charcot-Marie-Tooth type 2 F disease. Skeletal Radiol. (2012) 41:515–24. 10.1007/s00256-011-1199-y 21611841

[58] Yao L Yip AL Shrader JA Mesdaghinia S Volochayev R Jansen AV. Magnetic resonance measurement of muscle T2, fat-corrected T2 and fat fraction in the assessment of idiopathic inflammatory myopathies. Rheumatology (2016) 55:441–9. 10.1093/rheumatology/kev344 26412808 PMC4757924

[59] Figueroa-Bonaparte S Segovia S Llauger J Belmonte I Pedrosa I Alejaldre A. Muscle MRI findings in childhood/adult onset pompe disease correlate with muscle function. PLoS ONE (2016) 11:e0163493. 10.1371/journal.pone.0163493 27711114 PMC5053479

[60] Carlier PG Azzabou N de Sousa PL Hicks A Boisserie JM Amadon A. Skeletal muscle quantitative nuclear magnetic resonance imaging follow-up of adult Pompe patients. J Inherit Metab Dis. (2015) 38:565–72. 10.1007/s10545-015-9825-9 25749708 PMC4432102

[61] Figueroa-Bonaparte S Llauger J Segovia S Belmonte I Pedrosa I Montiel E. Quantitative muscle MRI to follow up late onset Pompe patients: a prospective study. Sci Rep. (2018) 8:10898. 10.1038/s41598-018-29170-7 30022036 PMC6052002

[62] Schlaeger S Klupp E Weidlich D Cervantes B Foreman SC Deschauer M. T2-weighted dixon turbo spin echo for accelerated simultaneous grading of whole-body skeletal muscle fat infiltration and edema in patients with neuromuscular diseases. J Comput Assist Tomogr. (2018) 42:574–9. 10.1097/RCT.0000000000000723 29613984

[63] Reeder SB Pineda AR Wen Z Shimakawa A Yu H Brittain JH. Iterative decomposition of water and fat with echo asymmetry and least-squares estimation (IDEAL): application with fast spin-echo imaging. Magn Reson Med. (2005) 54:636–44. 10.1002/mrm.20624 16092103

[64] Pineda AR Reeder SB Wen Z Pelc NJ. Cramér-Rao bounds for three-point decomposition of water and fat. Magn Reson Med. (2005) 54:625–35. 10.1002/mrm.20623 16092102

[65] Patten C Meyer RA Fleckenstein JL. T2 mapping of muscle. Semin Musculoskelet Radiol. (2003) 7:297–305. 10.1055/s-2004-815677 14735428

[66] Mercuri E Pichiecchio A Allsop J Messina S Pane M Muntoni F. Muscle MRI in inherited neuromuscular disorders: past, present, and future. J Magn Reson Imaging (2007) 25:433–40. 10.1002/jmri.20804 17260395

[67] Phoenix J Betal D Roberts N Helliwell TR Edwards RHT. Objective quantification of muscle and fat in human dystrophic muscle by magnetic resonance image analysis. Muscle Nerve (1996) 19:302–10. 8606693 10.1002/(SICI)1097-4598(199603)19:3<302::AID-MUS4>3.0.CO;2-H

[68] Sesto ME Chourasia AO Block WF Radwin RG. Mechanical and magnetic resonance imaging changes following eccentric or concentric exertions. Clin Biomech. (2008) 23:961–8. 10.1016/j.clinbiomech.2008.03.068 18485551 PMC2581652

[69] Varghese J Scandling D Joshi R Aneja A Craft J Raman SV Rapid assessment of quantitative T 1, T 2 and T 2 ^*^ in lower extremity muscles in response to maximal treadmill exercise. NMR Biomed. (2015) 28:998–1008. 10.1002/nbm.3332 26123219 PMC4524289

[70] Kim HK Laor T Horn PS Wong B. Quantitative assessment of the T2 relaxation time of the gluteus muscles in children with duchenne muscular dystrophy: a comparative study before and after steroid treatment. Korean J Radiol. (2010) 11:304–11. 10.3348/kjr.2010.11.3.304 20461184 PMC2864857

[71] Kim HK Laor T Horn PS Racadio JM Wong B Dardzinski BJ. T2 mapping in Duchenne muscular dystrophy: distribution of disease activity and correlation with clinical assessments. Radiology (2010) 255:899–908. 10.1148/radiol.10091547 20501727

[72] Hiba B Richard N Hébert LJ Coté C Nejjari M Vial C. Quantitative assessment of skeletal muscle degeneration in patients with myotonic dystrophy type 1 using MRI. J Magn Reson Imaging (2012) 35:678–85. 10.1002/jmri.22849 22069222

[73] Maillard SM Jones R Owens C Pilkington C Woo P Wedderburn LR. Quantitative assessment of MRI T2 relaxation time of thigh muscles in juvenile dermatomyositis. Rheumatology (2004) 43:603–8. 10.1093/rheumatology/keh130 14983103

[74] Mankodi A Azzabou N Bulea T Reyngoudt H Shimellis H Ren Y. Skeletal muscle water T2 as a biomarker of disease status and exercise effects in patients with Duchenne muscular dystrophy. Neuromusc Disord. (2017) 27:705–14. 10.1016/j.nmd.2017.04.008 28601553 PMC5538585

[75] Carlier PG. Global T2 versus water T2 in NMR imaging of fatty infiltrated muscles: different methodology, different information and different implications. Neuromusc Disord. (2014) 24:390–2. 10.1016/j.nmd.2014.02.009 24656605

[76] Huang Y Majumdar S Genant HK Chan WP Sharma KR Yu P. Quantitative MR relaxometry study of muscle composition and function in Duchenne muscular dystrophy. J Magn Reson Imaging (1994) 4:59–64. 10.1002/jmri.1880040113 8148557

[77] Arpan I Forbes SC Lott DJ Senesac CR Daniels MJ Triplett WT T2 mapping provides multiple approaches for the characterization of muscle involvement in neuromuscular diseases: a cross-sectional study of lower leg muscles in 5-15-year-old boys with Duchenne muscular dystrophy. NMR Biomed. (2013) 26:320–8. 10.1002/nbm.2851 23044995 PMC3573223

[78] Garrood P Hollingsworth KG Eagle M Aribisala BS Birchall D Bushby K. MR imaging in Duchenne muscular dystrophy: quantification of T1-weighted signal, contrast uptake, and the effects of exercise. J Magn Reson Imaging (2009) 30:1130–8. 10.1002/jmri.21941 19856446

[79] Forbes SC Willcocks RJ Triplett WT Rooney WD Lott DJ Wang DJ. Magnetic resonance imaging and spectroscopy assessment of lower extremity skeletal muscles in boys with duchenne muscular dystrophy: a multicenter cross sectional study. PLoS ONE (2014) 9:e106435. 10.1371/journal.pone.0106435 25203313 PMC4159278

[80] Johnston JH Kim HK Merrow AC Laor T Serai S Horn PS. Quantitative skeletal muscle MRI: part 1, derived T2 fat map in differentiation between boys with duchenne muscular dystrophy and healthy boys. Am J Roentgenol. (2015) 205:W207–15. 10.2214/AJR.14.13754 26204309

[81] Kim HK Serai S Lindquist D Merrow AC Horn PS Kim DH. Quantitative skeletal muscle MRI: part 2, MR spectroscopy and T2 relaxation time mapping- comparison between boys with duchenne muscular dystrophy and healthy boys. Am J Roentgenol. (2015) 205:W216–23. 10.2214/AJR.14.13755 26204310

[82] Gaur L Hanna A Bandettini WP Fischbeck KH Arai AE Mankodi A. Upper arm and cardiac magnetic resonance imaging in Duchenne muscular dystrophy. Ann Clin Transl Neurol. (2016) 3:948–55. 10.1002/acn3.367 28097207 PMC5224820

[83] Willcocks RJ Arpan IA Forbes SC Lott DJ Senesac CR Senesac E. Longitudinal measurements of MRI-T2 in boys with Duchenne muscular dystrophy: effects of age and disease progression. Neuromusc Disord. (2014) 24:393–401. 10.1016/j.nmd.2013.12.012 24491484 PMC4277599

[84] Barnard AM Willcocks RJ Finanger EL Daniels MJ Triplett WT Rooney WD. Skeletal muscle magnetic resonance biomarkers correlate with function and sentinel events in Duchenne muscular dystrophy. PLoS ONE (2018) 13:e0194283. 10.1371/journal.pone.0194283 29554116 PMC5858773

[85] Hooijmans MT Niks EH Burakiewicz J Verschuuren JJGM Webb AG Kan HE Elevated phosphodiester and T 2 levels can be measured in the absence of fat infiltration in Duchenne muscular dystrophy patients. NMR Biomed. (2017) 30:e3667 10.1002/nbm.3667 27859827

[86] Wokke BH Van Den Bergen JC Hooijmans MT Verschuuren JJ Niks EH Kan HE T2 relaxation times are increased in Skeletal muscle of DMD but not BMD patients. Muscle Nerve (2016) 53:38–43. 10.1002/mus.24679 25847364

[87] Yao L Gai N. Fat-corrected T2 measurement as a marker of active muscle disease in inflammatory myopathy. Am J Roentgenol. (2012) 198:W475–81. 10.2214/AJR.11.7113 22528929

[88] Carlier PG Azzabou N de Sousa PL Florkin B Deprez E Romero NB P.14.4 Diagnostic role of quantitative NMR imaging exemplified by 3 cases of juvenile dermatomyositis. Neuromusc Disord. (2013) 23:814 10.1016/j.nmd.2013.06.612

[89] Park JH Vital TL Ryder NM Hernanz-Schulman M Leon Partain C Price RR. Magnetic resonance imaging and p-31 magnetic resonance spectroscopy provide unique quantitative data useful in the longitudinal management of patients with dermatomyositis. Arthritis Rheum. (1994) 37:736–46. 10.1002/art.1780370519 8185702

[90] Park JH Olsen NJ Jr King L Vital T Buse R Kari S. Use of magnetic resonance imaging and p-31 magnetic resonance spectroscopy to detect and quantify muscle dysfunction in the amyopathic and myopathic variants of dermatomyositis. Arthritis Rheum. (1995) 38:68–77. 10.1002/art.1780380111 7818575

[91] Park JH Vansant JP Kumar NG Gibbs SJ Curvin MS Price RR. Dermatomyositis: correlative MR imaging and P-31 MR spectroscopy for quantitative characterization of inflammatory disease. Radiology (1990) 177:473–9. 10.1148/radiology.177.2.2217788 2217788

[92] Murphy WA Totty WG Carroll JE. MRI of normal and pathologic skeletal muscle. Am J Roentgenol. (1986) 146:565–74. 10.2214/ajr.146.3.565 3484872

[93] Fan RH Does MD. Compartmental relaxation and diffusion tensor imaging measurements *in vivo* in λ-carrageenan-induced edema in rat skeletal muscle. NMR Biomed. (2008) 21:566–73. 10.1002/nbm.1226 18041804 PMC2694448

[94] Bulluck H Maestrini V Rosmini S Abdel-Gadir A Treibel TA Castelletti S. Myocardial T1 Mapping. Circ J. (2015) 79:487–94. 10.1253/circj.CJ-15-0054 25746524

[95] Li K Dortch RD Welch EB Bryant ND Buck AKW Towse TF. Multi-parametric MRI characterization of healthy human thigh muscles at 3.0 T - relaxation, magnetization transfer, fat/water, and diffusion tensor imaging. NMR Biomed. (2014) 27:1070–84. 10.1002/nbm.3159 25066274 PMC4153695

[96] Leporq B Le Troter A Le Fur Y Salort-Campana E Guye M Beuf O Combined quantification of fatty infiltration, T1-relaxation times and T2^*^-relaxation times in normal-appearing skeletal muscle of controls and dystrophic patients. Magn Reson Mater Phys Biol Med. (2017) 30:407 10.1007/s10334-017-0616-1 28332039

[97] Marty B Coppa B Carlier PG. Monitoring skeletal muscle chronic fatty degenerations with fast T1-mapping. Eur Radiol. (2018) 28:4662–8. 10.1007/s00330-018-5433-z 29713767

[98] Qi J Olsen NJ Price RR Winston JA Park JH. Diffusion-weighted imaging of inflammatory myopathies: polymyositis and dermatomyositis. J Magn Reson Imaging (2008) 27:212–7. 10.1002/jmri.21209 18022843

[99] Ai T Yu K Gao L Zhang P Goerner F Runge VM. Diffusion tensor imaging in evaluation of thigh muscles in patients with polymyositis and dermatomyositis. Br J Radiol. (2014) 87:20140261. 10.1259/bjr.20140261 25183381 PMC4207168

[100] Oppenheim C Ducreux D Rodrigo S Hodel J Tourdias T Charbonneau F. Diffusion tensor imaging and tractography of the brain and spinal cord. J Radiol. (2007) 88:510–20. 10.1016/S0221-0363(07)89850-7 17457261

[101] Budzik J-F Balbi V Verclytte S Pansini V Le Thuc V Cotten A. Diffusion tensor imaging in musculoskeletal disorders. RadioGraph. (2014) 34:E56–72. 10.1148/rg.343125062 24819802

[102] Chianca V Albano D Messina C Cinnante CM Triulzi FM Sardanelli F. Diffusion tensor imaging in the musculoskeletal and peripheral nerve systems: from experimental to clinical applications. Eur Radiol Exp. (2017) 1:12. 10.1186/s41747-017-0018-1 29708174 PMC5909344

[103] Hooijmans MT Damon BM Froeling M Versluis MJ Burakiewicz J Verschuuren JJGM. Evaluation of skeletal muscle DTI in patients with duchenne muscular dystrophy. NMR Biomed. (2015) 28:1589–97. 10.1002/nbm.3427 26449628 PMC4670831

[104] Li GD Liang YY Xu P Ling J Chen YM. Diffusion-tensor imaging of thigh muscles in Duchenne muscular dystrophy: correlation of apparent diffusion coefficient and fractional anisotropy values with fatty infiltration. Am J Roentgenol. (2016) 206:867–70. 10.2214/AJR.15.15028 26866848

[105] Ponrartana S Ramos-Platt L Wren TAL Hu HH Perkins TG Chia JM. Effectiveness of diffusion tensor imaging in assessing disease severity in Duchenne muscular dystrophy: preliminary study. Pediatr Radiol. (2015) 45:582–9. 10.1007/s00247-014-3187-6 25246097

[106] Williams SE Heemskerk AM Welch EB Li K Damon BM Park JH. Quantitative effects of inclusion of fat on muscle diffusion tensor MRI measurements. J Magn Reson Imaging (2013) 38:1292–7. 10.1002/jmri.24045 23418124 PMC3664247

[107] Damon BM Heemskerk AM Ding Z. Polynomial fitting of DT-MRI fiber tracts allows accurate estimation of muscle architectural parameters. Magn Reson Imaging (2012) 30:589–600. 10.1016/j.mri.2012.02.003 22503094 PMC3348398

[108] Damon BM Froeling M Buck AKW Oudeman J Ding Z Nederveen AJ. Skeletal muscle diffusion tensor-MRI fiber tracking: rationale, data acquisition and analysis methods, applications and future directions. NMR Biomed. (2017) 30:e3563. 10.1002/nbm.3563 27257975 PMC5136336

[109] Keller S Wang ZJ Aigner A Kim AC Golsari A Kooijman H. Diffusion tensor imaging of dystrophic skeletal muscle: comparison of two segmentation methods adapted to chemical-shift-encoded water-fat MRI. Clin Neuroradiol. (2018). 10.1007/s00062-018-0667-3. [Epub ahead of print]. 29392347

[110] Hori M Ishigame K Shiraga N Kumagai H Aoki S Araki T. Mean diffusivity, fractional anisotropy maps, and three-dimensional white-matter tractography by diffusion tensor imaging. Comparison between single-shot fast spin-echo and single-shot echo-planar sequences at 1.5 Tesla. Eur Radiol. (2008) 18:830–4. 10.1007/s00330-007-0805-9 17999065

[111] Le Bihan D Breton E Lallemand D Grenier P Cabanis E Laval-Jeantet M MR imaging of intravoxel incoherent motions: application to diffusion and perfusion in neurologic disorders. Radiology (1986) 161:401–7. 10.1148/radiology.161.2.3763909 3763909

[112] Hiepe P Gussew A Rzanny R Anders C Walther M Scholle HC Interrelations of muscle functional MRI, diffusion-weighted MRI and 31P-MRS in exercised lower back muscles. NMR Biomed. (2014) 27:958–70. 10.1002/nbm.3141 24953438

[113] Filli L Winklhofer S Andreisek G Del Grande F. Imaging of myopathies. Radiol Clin North Am. (2017) 55:1055–70. 10.1016/j.rcl.2017.04.010 28774448

[114] Karampinos DC King KF Sutton BP Georgiadis JG. Intravoxel partially coherent motion technique: characterization of the anisotropy of skeletal muscle microvasculature. J Magn Reson Imaging (2010) 31:942–53. 10.1002/jmri.22100 20373440

[115] Sigmund EE Baete SH Luo T Patel K Wang D Rossi I. MRI assessment of the thigh musculature in dermatomyositis and healthy subjects using diffusion tensor imaging, intravoxel incoherent motion and dynamic DTI. Eur Radiol. (2018) 28:5304–15. 10.1007/s00330-018-5458-3 29869178 PMC11980643

[116] Ran J Liu Y Sun D Morelli J Zhang P Wu G. The diagnostic value of biexponential apparent diffusion coefficients in myopathy. J Neurol. (2016) 263:1296–302. 10.1007/s00415-016-8139-7 27142711

[117] Henkelman RM Stanisz GJ Graham SJ. Magnetization transfer in MRI: a review. NMR Biomed. (2001) 14:57–64. 10.1002/nbm.683 11320533

[118] Li K Dortch RD Kroop SF Huston JW Gochberg DF Park JH. A rapid approach for quantitative magnetization transfer imaging in thigh muscles using the pulsed saturation method. Magn Reson Imaging (2015) 33:709–17. 10.1016/j.mri.2015.03.003 25839394 PMC4755108

[119] Morrow JM Sinclair CDJ Fischmann A Reilly MM Hanna MG Yousry TA. Reproducibility, and age, body-weight and gender dependency of candidate skeletal muscle MRI outcome measures in healthy volunteers. Eur Radiol. (2014) 24:1610–20. 10.1007/s00330-014-3145-6 24748539 PMC4046083

[120] Schwenzer NF Martirosian P Machann J Schraml C Steidle G Claussen CD. Aging effects on human calf muscle properties assessed by MRI at 3 Tesla. J Magn Reson Imaging (2009) 29:1346–54. 10.1002/jmri.21789 19472391

[121] Sinclair CDJ Morrow JM Miranda MA Davagnanam I Cowley PC Mehta H. Skeletal muscle MRI magnetisation transfer ratio reflects clinical severity in peripheral neuropathies. J Neurol Neurosurg Psychiatry (2012) 83:29–32. 10.1136/jnnp.2011.246116 21613652

[122] Sinclair CDJ Samson RS Thomas DL Weiskopf N Lutti A Thornton JS. Quantitative magnetization transfer in *in vivo* healthy human skeletal muscle at 3 T. Magn Reson Med. (2010) 64:1739–48. 10.1002/mrm.22562 20665899 PMC3077519

[123] Bryant ND Li K Does MD Barnes S Gochberg DF Yankeelov TE. Multi-parametric MRI characterization of inflammation in murine skeletal muscle. NMR Biomed. (2014) 27:716–25. 10.1002/nbm.3113 24777935 PMC4134016

[124] Kusmia S Eliav U Navon G Guillot G. DQF-MT MRI of connective tissues: application to tendon and muscle. Magn Reson Mater Physics, Biol Med. (2013) 26:203–14. 10.1007/s10334-012-0346-3 23001199

[125] Boesch C. Musculoskeletal spectroscopy. J Magn Reson Imaging (2007) 25:321–38. 10.1002/jmri.20806 17260389

[126] Edwards RH Wilkie DR Joan Dawson M Gordon RE Shaw D. Clinical use of nuclear magnetic resonance in the investigation of myopathy. Lancet (1982) 1:725–31. 10.1016/S0140-6736(82)92635-6 6122019

[127] Barbiroli B Funicello R Ferlini A Montagna P Zaniol P. Muscle energy metabolism in female DMD/BMD carriers: a 31P-MR spectroscopy study. Muscle Nerve (1992) 15:344–8. 10.1002/mus.880150313 1557082

[128] Kemp GJ Taylor DJ Dunn JF Frostick SP Radda GK. Cellular energetics of dystrophic muscle. J Neurol Sci. (1993) 116:201–6. 10.1016/0022-510X(93)90326-T 8393092

[129] Tonon C Gramegna LL Lodi R. Magnetic resonance imaging and spectroscopy in the evaluation of neuromuscular disorders and fatigue. Neuromusc Disord. (2012) 22(Suppl. 3):S187–91. 10.1016/j.nmd.2012.10.008 23182637

[130] Burt CT Glonek T Bárány M. Phosphorus-31 nuclear magnetic resonance detection of unexpected phosphodiesters in muscle. Biochemistry (1976) 15:4850–3. 990247 10.1021/bi00667a015

[131] Ruiz-Cabello J Cohen JS. Phospholipid metabolites as indicators of cancer cell function. NMR Biomed. (1992) 5:226–33. 10.1002/nbm.1940050506 1449961

[132] Amarteifio E Nagel AM Kauczor H-U Weber M-A. Functional imaging in muscular diseases. Insights Imaging (2011) 2:609–19. 10.1007/s13244-011-0111-6 22347980 PMC3259416

[133] Wokke BH Hooijmans MT van den Bergen JC Webb AG Verschuuren JJ Kan HE. Muscle MRS detects elevated PDE/ATP ratios prior to fatty infiltration in Becker muscular dystrophy. NMR Biomed. (2014) 27:1371–7. 10.1002/nbm.3199 25196814

[134] Hooijmans MT Doorenweerd N Baligand C Verschuuren JJGM Ronen I Niks EH. Spatially localized phosphorous metabolism of skeletal muscle in Duchenne muscular dystrophy patients: 24–Month follow-up. PLoS ONE (2017) 12:e0182086. 10.1371/journal.pone.0182086 28763477 PMC5538641

[135] Torriani M Townsend E Thomas BJ Bredella MA Ghomi RH Tseng BS. Lower leg muscle involvement in Duchenne muscular dystrophy: an MR imaging and spectroscopy study. Skeletal Radiol. (2012) 41:437–45. 10.1007/s00256-011-1240-1 21800026 PMC3713639

[136] Kan HE Klomp DWJ Wong CS Boer VO Webb AG Luijten PR. *in vivo* 31P MRS detection of an alkaline inorganic phosphate pool with short T1 in human resting skeletal muscle. NMR Biomed. (2010) 23:995–1000. 10.1002/nbm.1517 20878975 PMC3856567

[137] Hsieh T-J Jaw T-S Chuang H-Y Jong Y-J Liu G-C Li C-W. Muscle metabolism in Duchenne muscular dystrophy assessed by *in vivo* proton magnetic resonance spectroscopy. J Comput Assist Tomogr. (2009) 33:150–4. 10.1097/RCT.0b013e318168f735 19188804

[138] Younkin DP Berman P Sladky J Chee C Bank W Chance B. 31P NMR studies in Duchenne muscular dystrophy: age-related metabolic changes. Neurology (1987) 37:165–9. 10.1212/WNL.37.1.165 3796830

[139] Ikehira H Nishikawa S Matsumura K Hasegawa T Arimizu N Tateno Y. The functional staging of Duchenne muscular dystrophy using *in vivo* 31P MR spectroscopy. Radiat Med. (1995) 13:63–5. 7667509

[140] Wary C Azzabou N Giraudeau C Le Louër J Montus M Voit T. Quantitative NMRI and NMRS identify augmented disease progression after loss of ambulation in forearms of boys with Duchenne muscular dystrophy. NMR Biomed. (2015) 28:1150–62. 10.1002/nbm.3352 26215733

[141] Barbiroli B Funicello R Iotti S Montagna P Ferlini A Zaniol P. 31P-NMR spectroscopy of skeletal muscle in Becker dystrophy and DMD/BMD carriers. Altered rate of phosphate transport. J Neurol Sci. (1992) 109:188–95. 10.1016/0022-510X(92)90167-J 1634901

[142] Lodi R Kemp GJ Muntoni F Thompson CH Rae C Taylor J. Reduced cytosolic acidification during exercise suggests defective glycolytic activity in skeletal muscle of patients with Becker muscular dystrophy. An *in vivo* 31P magnetic resonance spectroscopy study. Brain (1999) 122:121–30. 10.1093/brain/122.1.121 10050900

[143] Argov Z Lofberg M Arnold DL. Insights into muscle diseases gained by phosphorus magnetic resonance spectroscopy. Muscle Nerve (2000) 23:1316–34. 10951434 10.1002/1097-4598(200009)23:9<1316::aid-mus2>3.0.co;2-i

[144] Tosetti M Linsalata S Battini R Volpi L Cini C Presciutti O. Muscle metabolic alterations assessed by 31-phosphorus magnetic resonance spectroscopy in mild Becker muscular dystrophy. Muscle Nerve (2011) 44:816–9. 10.1002/mus.22181 21952990

[145] Lodi R Muntoni F Taylor J Kumar S Sewry CA Blamire A. Correlative MR imaging and 31P-MR spectroscopy study in sarcoglycan deficient limb girdle muscular dystrophy. Neuromusc Disord. (1997) 7:505–11. 10.1016/S0960-8966(97)00108-9 9447608

[146] Barbiroli B McCully KK Iotti S Lodi R Zaniol P Chance B. Further impairment of muscle phosphate kinetics by lengthening exercise in DMD/BMD carriers. An *in vivo* 31P-NMR spectroscopy study. J Neurol Sci. (1993) 119:65–73. 10.1016/0022-510X(93)90192-2 8246012

[147] Gramegna LL Giannoccaro MP Manners DN Testa C Zanigni S Evangelisti S. Mitochondrial dysfunction in myotonic dystrophy type 1. Neuromusc Disord. (2018) 28:144–9. 10.1016/j.nmd.2017.10.007 29289451

[148] Banerjee B Sharma U Balasubramanian K Kalaivani M Kalra V Jagannathan NR. Effect of creatine monohydrate in improving cellular energetics and muscle strength in ambulatory Duchenne muscular dystrophy patients: a randomized, placebo-controlled 31P MRS study. Magn Reson Imaging (2010) 28:698–707. 10.1016/j.mri.2010.03.008 20395096

[149] Kan HE Klomp DWJ Wohlgemuth M Van Loosbroek-Wagemans I Van Engelen BGM Padberg GW. Only fat infiltrated muscles in resting lower leg of FSHD patients show disturbed energy metabolism. NMR Biomed. (2010) 23:563–8. 10.1002/nbm.1494 20175146

[150] Janssen BH Voet NBM Nabuurs CI Kan HE De Rooy JWJ Geurts AC. Distinct disease phases in muscles of facioscapulohumeral dystrophy patients identified by MR detected fat infiltration. PLoS ONE (2014) 9:e85416. 10.1371/journal.pone.0085416 24454861 PMC3891814

[151] Okuma H Kurita D Ohnuki T Haida M Shinohara Y. Muscle metabolism in patients with polymyositis simultaneously evaluated by using 31P-magnetic resonance spectroscopy and near-infrared spectroscopy. Int J Clin Pract. (2006) 61:684–9. 10.1111/j.1742-1241.2006.00968.x 16889559

[152] Lodi R Taylor DJ Tabrizi SJ Hilton-Jones D Squier MV Seller A. Normal *in vivo* skeletal muscle oxidative metabolism in sporadic inclusion body myositis assessed by 31P-magnetic resonance spectroscopy. Brain (1998) 121:2119–26. 10.1093/brain/121.11.2119 9827771

[153] Fischer MA Nanz D Shimakawa A Schirmer T Guggenberger R Chhabra A. Quantification of muscle fat in patients with low back pain: comparison of multi-echo MR imaging with single-voxel MR spectroscopy. Radiology (2013) 266:555–63. 10.1148/radiol.12120399 23143025

[154] Reeder SB Cruite I Hamilton G Sirlin CB Quantitative assessment of liver fat with magnetic resonance imaging and spectroscopy. J Magn Reson Imaging (2011)34:729–49. 10.1002/jmri.22580 22025886 PMC3177109

[155] Marden FA Connolly AM Siegel MJ Rubin DA. Compositional analysis of muscle in boys with Duchenne muscular dystrophy using MR imaging. Skeletal Radiol. (2005) 34:140–8. 10.1007/s00256-004-0825-3 15538561

[156] Hsieh T-J Wang C-K Chuang H-Y Jong Y-J Li C-W Liu G-C. *in vivo* proton magnetic resonance spectroscopy assessment for muscle metabolism in neuromuscular diseases. J Pediatr. (2007) 151:319–21. 10.1016/j.jpeds.2007.05.026 17719948

[157] Lott DJ Forbes SC Mathur S Germain SA Senesac CR Lee Sweeney H. Assessment of intramuscular lipid and metabolites of the lower leg using magnetic resonance spectroscopy in boys with Duchenne muscular dystrophy. Neuromusc Disord. (2014) 24:574–82. 10.1016/j.nmd.2014.03.013 24798221 PMC4142654

[158] Subhawong TK Wang X MacHado AJ Mammen AL Christopher-Stine L Barker PB. 1H magnetic resonance spectroscopy findings in idiopathic inflammatory myopathies at 3 T: feasibility and first results. Invest Radiol. (2013) 48:509–16. 10.1097/RLI.0b013e3182823562 23563194

[159] Koch KS Leffert HL. Increased sodium ion influx is necessary to initiate rat hepatocyte proliferation. Cell (1979) 18:153–63. 10.1016/0092-8674(79)90364-7 509519

[160] Cameron IL Smith NKR Pool TB Sparks RL. Intracellular concentration of sodium and other elements as related to mitogenesis and oncogenesis *in vivo*. Cancer Res. (1980) 40:1493–500. 7370987

[161] Kushnir T Knubovets T Itzchak Y Eliav U Sadeh M Rapoport L. *in vivo* 23Na NMR studies of myotonic dystrophy. Magn Reson Med. (1997) 37:192–6. 10.1002/mrm.1910370209 9001142

[162] Constantinides CD Gillen JS Boada FE Pomper MG Bottomley PA. Human skeletal muscle: sodium mr imaging and quantification—potential applications in exercise and disease. Radiology (2000) 216:559–68. 10.1148/radiology.216.2.r00jl46559 10924586

[163] Madelin G Regatte RR. Biomedical applications of sodium MRI *in vivo*. J Magn Reson Imaging (2013) 38:511–29. 10.1002/jmri.24168 23722972 PMC3759542

[164] Weber MA Nagel AM Jurkat-Rott K Lehmann-Horn F. Sodium (23Na) MRI detects elevated muscular sodium concentration in Duchenne muscular dystrophy. Neurology (2011) 77:2017–24. 10.1212/WNL.0b013e31823b9c78 22116947

[165] Glemser PA Jaeger H Nagel AM Ziegler AE Simons D Schlemmer H. 23Na MRI and myometry to compare eplerenone vs. glucocorticoid treatment in Duchenne dystrophy. Acta Myol. (2017) 36:2–13. 28690388 PMC5479105

[166] Shulman GI Rothman DL Jue T Stein P DeFronzo RA Shulman RG. Quantitation of muscle glycogen synthesis in normal subjects and subjects with non-insulin-dependent diabetes by 13C nuclear magnetic resonance spectroscopy. N Engl J Med. (1990) 322:223–8. 10.1056/NEJM199001253220403 2403659

[167] Jehenson P Duboc D Bloch G Fardeau M Syrota A. Diagnosis of muscular glycogenosis by *in vivo* natural abundance13C NMR spectroscopy. Neuromusc Disord. (1991) 1:99–101. 10.1016/0960-8966(91)90056-X 1822788

[168] Heinicke K Dimitrov IE Romain N Cheshkov S Ren J Malloy CR. Reproducibility and absolute quantification of muscle glycogen in patients with glycogen storage disease by 13C NMR spectroscopy at 7 tesla. PLoS ONE (2014) 9:e108706. 10.1371/journal.pone.0108706 25296331 PMC4189928

[169] Aquaro GD Menichetti L. Hyperpolarized 13C-magnetic resonance spectroscopy are we ready for metabolic imaging? Circ Cardiovasc Imaging (2014) 7:854–6. 10.1161/CIRCIMAGING.114.002648 25406195

[170] Wary C Laforêt P Eymard B Fardeau M Leroy-Willig A Bassez. Evaluation of muscle glycogen content by 13C NMR spectroscopy in adult-onset acid maltase deficiency. Neuromusc Disord. (2003) 13:545–53. 10.1016/S0960-8966(03)00069-5 12921791

[171] Mariappan YK Glaser KJ Ehman RL. Magnetic resonance elastography: a review. Clin Anat. (2010) 23:497–511. 10.1002/ca.21006 20544947 PMC3066083

[172] Glaser KJ Manduca A Ehman RL. Review of MR elastography applications and recent developments. J Magn Reson Imaging (2012) 36:757–74. 10.1002/jmri.23597 22987755 PMC3462370

[173] Song J Kwon OI Seo JK Anisotropic elastic moduli reconstruction in transversely isotropic model using MRE. Inverse Probl. (2012) 28:115003 10.1088/0266-5611/28/11/115003

[174] Ringleb SI Bensamoun SF Chen Q Manduca A An KN Ehman RL. Applications of magnetic resonance elastography to healthy and pathologic skeletal muscle. J Magn Reson Imaging (2007) 25:301–9. 10.1002/jmri.20817 17260391

[175] Hong SH Hong S-J Yoon J-S Oh C-H Cha JG Kim HK. Magnetic resonance elastography (MRE) for measurement of muscle stiffness of the shoulder: feasibility with a 3 T MRI system. Acta Radiol. (2015) 57:1099–106. 10.1177/0284185115571987 25711231

[176] Basford JR Jenkyn TR An K-N Ehman RL Heers G Kaufman KR. Evaluation of healthy and diseased muscle with magnetic resonance elastography. Arch Phys Med Rehabil. (2002) 83:1530–6. 10.1053/apmr.2002.35472 12422320

[177] Guermazi A Roemer FW Robinson P Tol JL Regatte RR Crema MD. Imaging of muscle injuries in sports medicine: sports imaging series. Radiology (2017)282:646–63. 10.1148/radiol.2017160267 28218878

[178] Lacourpaille L Hug F Guével A Péréon Y Magot A Hogrel J-Y. Non-invasive assessment of muscle stiffness in patients with duchenne muscular dystrophy. Muscle Nerve (2015) 51:284–6. 10.1002/mus.24445 25187068

[179] McCullough MB Domire ZJ Reed AM Amin S Ytterberg SR Chen Q. Evaluation of muscles affected by myositis using magnetic resonance elastography. Muscle Nerve (2011) 43:585–90. 10.1002/mus.21923 21319167 PMC3059125

[180] Brandenburg JE Eby SF Song P Zhao H Brault JS Chen S. Ultrasound elastography: the new frontier in direct measurement of muscle stiffness. Arch Phys Med Rehabil. (2014) 95:2207–19. 10.1016/j.apmr.2014.07.007 25064780 PMC4254343

[181] Drakonaki EE Allen GM Wilson DJ. Ultrasound elastography for musculoskeletal applications. Br J Radiol. (2012) 85:1435–45. 10.1259/bjr/93042867 23091287 PMC3500785

[182] Berko NS Fitzgerald EF Amaral TD Payares M Levin TL. Ultrasound elastography in children: establishing the normal range of muscle elasticity. Pediatr Radiol. (2014) 44:158–63. 10.1007/s00247-013-2793-z 24104402

[183] Dastgir J Vuillerot C Nguyen D Yang K Auh S Donkervoort S Acoustic radiation force impulse imaging for the longitudinal assessment of muscle tissue stiffness in collagen 6 myopathy and LAMA2 related muscular dystrophy. Neuromusc Disord. (2014) 24:908 10.1016/j.nmd.2014.06.379

[184] Dastgir J Vuillerot C Harrison K Poon A Donkervoort S Leach M Acoustic radiation force impulse imaging for the differentiation of muscle tissue stiffness in neuromuscular disorders. Neuromusc Disord. (2013) 23:811 10.1016/j.nmd.2013.06.603

[185] Zaidman CM Malkus EC Connolly AM. Muscle ultrasound quantifies disease progression over time in infants and young boys with Duchenne muscular dystrophy. Muscle Nerve (2015) 52:334–8. 10.1002/mus.24609 25704979 PMC5931214

[186] Pichiecchio A Alessandrino F Bortolotto C Cerica A Rosti C Raciti MV. Muscle ultrasound elastography and MRI in preschool children with Duchenne muscular dystrophy. Neuromusc Disord. (2018) 28:476–83. 10.1016/j.nmd.2018.02.007 29661643

[187] Damon BM Wadington MC Hornberger JL Lansdown DA. Absolute and relative contributions of BOLD effects to the muscle functional MRI signal intensity time course: effect of exercise intensity. Magn Reson Med. (2007) 58:335–45. 10.1002/mrm.21319 17654591 PMC4440487

[188] Noseworthy MD Davis AD Elzibak AH. Advanced MR imaging techniques for skeletal muscle evaluation. Semin Musculoskelet Radiol. (2010) 14:257–68. 10.1055/s-0030-1253166 20486033

[189] Towse TF Slade JM Ambrose JA DeLano MC Meyer RA. Quantitative analysis of the postcontractile blood-oxygenation-level-dependent (BOLD) effect in skeletal muscle. J Appl Physiol. (2011) 111:27–39. 10.1152/japplphysiol.01054.2009 21330621 PMC3137544

[190] Towse TF Elder CP Bush EC Klockenkemper SW Bullock JT Dortch RD. Post-contractile BOLD contrast in skeletal muscle at 7 T reveals inter-individual heterogeneity in the physiological responses to muscle contraction. NMR Biomed. (2016) 29:1720–8. 10.1002/nbm.3593 27753155 PMC6594689

[191] Stacy MR Caracciolo CM Qiu M Pal P Varga T Constable RT. Comparison of regional skeletal muscle tissue oxygenation in college athletes and sedentary control subjects using quantitative BOLD MR imaging. Physiol Rep. (2016) 4:e12903. 10.14814/phy2.12903 27535483 PMC5002911

[192] Schulte A-C Aschwanden M Bilecen D. Calf Muscles at blood oxygen level–dependent mr imaging: aging effects at postocclusive reactive hyperemia. Radiology (2008) 247:482–9. 10.1148/radiol.2472070828 18372453

[193] Jacobi B Schulte AC Partovi S Michel S Karimi S Lyo JK. Alterations of skeletal muscle microcirculation detected by blood oxygenation level-dependent MRI in a patient with granulomatosis with polyangiitis. Rheumatology (2013) 52:579–81. 10.1093/rheumatology/kes176 22847680

[194] Caramia F Mainero C Gragnani F Tinelli E Fiorelli M Ceschin V. Functional MRI changes in the central motor system in myotonic dystrophy type 1. Magn Reson Imaging (2010) 28:226–34. 10.1016/j.mri.2009.07.006 19695817

[195] Raynaud JS Duteil S Vaughan JT Hennel F Wary C Leroy-Willig A. Determination of skeletal muscle perfusion using arterial spin labeling NMRI: validation by comparison with venous occlusion plethysmography. Magn Reson Med. (2001) 46:305–11. 10.1002/mrm.1192 11477634

[196] Decorte N Buehler T De Almeida Araujo EC Vignaud A Carlier PG Noninvasive estimation of oxygen consumption in human calf muscle through combined NMR measurements of ASL perfusion and T2 oxymetry. J Vasc Res. (2014) 51:360–8. 10.1159/000368194 25531648

[197] Boss A Martirosian P Claussen CD Schick F. Quantitative ASL muscle perfusion imaging using a FAIR-TrueFISP technique at 3.0 T. NMR Biomed. (2006) 19:125–32. 10.1002/nbm.1013 16404727

[198] Schewzow K Bernd Fiedler G Meyerspeer M Goluch S Laistler E Wolzt M. Dynamic ASL and T2*-weighted MRI in exercising calf muscle at 7 T-A feasibility study. Magn Reson Med. (2015) 73:1190–5. 10.1002/mrm.25242 24752959

[199] Andreisek G White LM Sussman MS Langer DL Patel C Su JW-S. T2^*^-weighted and arterial spin labeling MRI of calf muscles in healthy volunteers and patients with chronic exertional compartment syndrome: preliminary experience. AJR Am J Roentgenol. (2009) 193:W327–33. 10.2214/AJR.08.1579 19770303

[200] Pollak AW Meyer CH Epstein FH Jiji RS Hunter JR Dimaria JM. Arterial spin labeling MR imaging reproducibly measures peak-exercise calf muscle perfusion: a study in patients with peripheral arterial disease and healthy volunteers. JACC Cardiovasc Imaging (2012) 5:1224–30. 10.1016/j.jcmg.2012.03.022 23236972 PMC3531823

[201] Grözinger G Pohmann R Schick F Grosse U Syha R Brechtel K. Perfusion measurements of the calf in patients with peripheral arterial occlusive disease before and after percutaneous transluminal angioplasty using mr arterial spin labeling. J Magn Reson Imaging (2013) 40:980–7. 10.1002/jmri.24463 24243496

[202] Partovi S Karimi S Jacobi B Schulte AC Aschwanden M Zipp L. Clinical implications of skeletal muscle blood-oxygenation-level-dependent (BOLD) MRI. Magn Reson Mater Phys Biol Med. (2012) 25:251–61. 10.1007/s10334-012-0306-y 22374263

[203] Wattjes MP Fischer D Neuromuscular Imaging. New York, NY; Heidelberg; Dordrecht; London: Springer (2013). 10.1007/978-1-4614-6552-2

[204] Moon JC Messroghli DR Kellman P Piechnik SK Robson MD Ugander M. Myocardial T1 mapping and extracellular volume quantification: a Society for Cardiovascular Magnetic Resonance (SCMR) and CMR Working Group of the European Society of Cardiology consensus statement. J Cardiovasc Magn Reson. (2013) 15:92. 10.1186/1532-429X-15-92 24124732 PMC3854458

[205] Akbari A Rockel CP Kumbhare DA Noseworthy MD. Safe MRI-compatible electrical muscle stimulation (EMS) system. J Magn Reson Imaging (2016) 44:1530–8. 10.1002/jmri.25316 27185587

[206] Deligianni X Pansini M Garcia M Hirschmann A Schmidt-Trucksäss A Bieri O. Synchronous MRI of muscle motion induced by electrical stimulation. Magn Reson Med. (2017) 77:664–72. 10.1002/mrm.26154 26898990

[207] Nailon WH Texture analysis methods for medical image characterisation. In: Mao Y editor. Biomedical Imaging. London: IntechOpen (2010). p. 75–100. 10.5772/8912 Available online at: https://www.intechopen.com/journals/biomedical-imaging/texture-analysis-methods-for-medical-image-characterisation.

[208] Herlidou S Rolland Y Bansard JY Le Rumeur E de Certaines JD. Comparison of automated and visual texture analysis in MRI: characterization of normal and diseased skeletal muscle. Magn Reson Imaging (1999) 17:1393–7. 10.1016/S0730-725X(99)00066-1 10576724

[209] Scalco E Rancati T Pirovano I Mastropietro A Palorini F Cicchetti A. Texture analysis of T1-w and T2-w MR images allows a quantitative evaluation of radiation-induced changes of internal obturator muscles after radiotherapy for prostate cancer. Med Phys. (2018) 45:1518–28. 10.1002/mp.12798 29415344

[210] Mannil M Burgstaller JM Thanabalasingam A Winklhofer S Betz M Held U. Texture analysis of paraspinal musculature in MRI of the lumbar spine: analysis of the lumbar stenosis outcome study (LSOS) data. Skeletal Radiol. (2018) 47:947–54. 10.1007/s00256-018-2919-3 29497775

[211] Molinari F Caresio C Acharya UR Mookiah MRK Minetto MA. Advances in quantitative muscle ultrasonography using texture analysis of ultrasound images. Ultrasound Med Biol. (2015) 41:2520–32. 10.1016/j.ultrasmedbio.2015.04.021 26026375

[212] König T Steffen J Rak M Neumann G von Rohden L Tönnies KD. Ultrasound texture-based CAD system for detecting neuromuscular diseases. Int J Comput Assist Radiol Surg. (2015) 10:1493–503. 10.1007/s11548-014-1133-6 25451320

[213] Shklyar I Geisbush TR Mijialovic AS Pasternak A Darras BT Wu JS. Quantitative muscle ultrasound in Duchenne muscular dystrophy: a comparison of techniques. Muscle Nerve (2015) 51:207–13. 10.1002/mus.24296 24862337 PMC4241391

[214] Sogawa K Nodera H Takamatsu N Mori A Yamazaki H Shimatani Y. Neurogenic and myogenic diseases: quantitative texture analysis of muscle US data for differentiation. Radiology (2017) 283:492–8. 10.1148/radiol.2016160826 28156201

[215] Nyflot MJ Yang F Byrd D Bowen SR Sandison GA Kinahan PE. Quantitative radiomics: impact of stochastic effects on textural feature analysis implies the need for standards. J Med Imaging (2015) 2:041002. 10.1117/1.JMI.2.4.041002 26251842 PMC4524811

[216] De Certaines JD Larcher T Duda D Azzabou N Eliat P Escudero LM Application of texture analysis to muscle MRI: 1-What kind of information should be expected from texture analysis? EPJ Nonlinear Biomed Phys. (2015) 3:3 10.1140/epjnbp/s40366-015-0017-1

[217] Lerski RA de Certaines JD Duda D Klonowski W Yang G Coatrieux JL Application of texture analysis to muscle MRI: 2 – technical recommendations. EPJ Nonlinear Biomed Phys. (2015) 3:2 10.1140/epjnbp/s40366-015-0018-0

[218] Walker UA. Imaging tools for the clinical assessment of idiopathic inflammatory myositis. Curr Opin Rheumatol. (2008) 20:656–61. 10.1097/BOR.0b013e3283118711 18946324

[219] Ahmad N Welch I Grange R Hadway J Dhanvantari S Hill D. Use of imaging biomarkers to assess perfusion and glucose metabolism in the skeletal muscle of dystrophic mice. BMC Musculoskelet Disord. (2011) 12:127. 10.1186/1471-2474-12-127 21639930 PMC3141608

[220] Wattjes MP Nuclear medicine methods. In: Wattjes MP Fischer D editors. Neuromuscular Imaging. New York, NY; Heidelberg; Dordrecht; London: Springer (2013). p. 55–61. 10.1007/978-1-4614-6552-2_6

[221] Liau N Ooi C Reid C Kirkwood ID Bartholomeusz D. F-18 FDG PET/CT detection of mediastinal malignancy in a patient with dermatomyositis. Clin Nucl Med. (2007) 32:304–5. 10.1097/01.rlu.0000257282.76269.85 17413581

[222] Renard D Chiper L Collombier L Labauge P. Increased muscle FDG-PET uptake in dermatomyositis. J Neurol Neurosurg Psychiatry (2012) 83:487. 10.1136/jnnp-2011-301712 22180645

[223] Al-Nahhas A Jawad ASM. PET/CT imaging in inflammatory myopathies. Ann NY Acad Sci. (2011) 1228:39–45. 10.1111/j.1749-6632.2011.06016.x 21718321

[224] Tanaka S Ikeda K Uchiyama K Iwamoto T Sanayama Y Okubo A. [18F]Fdg uptake in proximal muscles assessed by pet/ct reflects both global and local muscular inflammation and provides useful information in the management of patients with polymyositis/dermatomyositis. Rheumatology (2013) 52:1271–8. 10.1093/rheumatology/ket112 23479721

[225] Tateyama M Fujihara K Misu T Arai A Kaneta T Aoki M. Clinical values of FDG PET in polymyositis and dermatomyositis syndromes: imaging of skeletal muscle inflammation. BMJ Open (2015) 5:e006763. 10.1136/bmjopen-2014-006763 25582454 PMC4298089

[226] Li Y Zhou Y Wang Q. Multiple values of 18F-FDG PET/CT in idiopathic inflammatory myopathy. Clin Rheumatol. (2017) 36:2297–305. 10.1007/s10067-017-3794-3 28831580

[227] Thøgersen KF Simonsen JA Hvidsten S Gerke O Jacobsen S Høilund-Carlsen PF. Quantitative 3D scintigraphy shows increased muscular uptake of pyrophosphate in idiopathic inflammatory myopathy. EJNMMI Res. (2017) 7:97. 10.1186/s13550-017-0348-2 29222707 PMC5722781

[228] Antoni G Lubberink M Estrada S Axelsson J Carlson K Lindsjo L. *In vivo* visualization of amyloid deposits in the heart with 11C-PIB and PET. J Nucl Med. (2013) 54:213–20. 10.2967/jnumed.111.102053 23238792

[229] Maetzler W Reimold M Schittenhelm J Vorgerd M Bornemann A Kötter I. Increased [11C]PIB-PET levels in inclusion body myositis are indicative of amyloid β deposition. J Neurol Neurosurg Psychiatry (2011) 82:1060–2. 10.1136/jnnp.2009.197640 20732867

[230] Andersen KF Jensen KE Loft A. PET/MR imaging in musculoskeletal disorders. PET Clin. (2016) 11:453–63. 10.1016/j.cpet.2016.05.007 27593249

[231] Jacobs MA Pan L Macura KJ. Whole-body diffusion-weighted and proton imaging: a review of this emerging technology for monitoring metastatic cancer. Semin Roentgenol. (2009) 44:111–22. 10.1053/j.ro.2009.01.003 19233086 PMC2955431

[232] Weckbach S Michaely HJ Stemmer A Schoenberg SO Dinter DJ. Comparison of a new whole-body continuous-table-movement protocol versus a standard whole-body MR protocol for the assessment of multiple myeloma. Eur Radiol. (2010) 20:2907–16. 10.1007/s00330-010-1865-9 20574630

[233] Karlsson A Rosander J Romu T Tallberg J Grönqvist A Borga M. Automatic and quantitative assessment of regional muscle volume by multi-atlas segmentation using whole-body water-fat MRI. J Magn Reson Imaging (2015) 41:1558–69. 10.1002/jmri.24726 25111561

[234] West J Leinhard OD Romu T Collins R Garratt S Bell JD. Feasibility of MR-based body composition analysis In large scale population studies. PLoS ONE (2016) 11:e0163332. 10.1371/journal.pone.0163332 27662190 PMC5035023

[235] Crawford RJ Filli L Elliott JM Nanz D Fischer MA Marcon M. Age- and level-dependence of fatty infiltration in lumbar paravertebral muscles of healthy volunteers. Am J Neuroradiol. (2016) 37:742–8. 10.3174/ajnr.A4596 26635285 PMC7960169

[236] Díaz-Manera J Alejaldre A González L Olivé M Gómez-Andrés D Muelas N. Muscle imaging in muscle dystrophies produced by mutations in the EMD and LMNA genes. Neuromusc Disord. (2016) 26:33–40. 10.1016/j.nmd.2015.10.001 26573435

[237] Tomas X Milisenda JC Garcia-Diez AI Prieto-Gonzalez S Faruch M Pomes J. Whole-body MRI and pathological findings in adult patients with myopathies. Skeletal Radiol. (2018). 10.1007/s00256-018-3107-1. [Epub ahead of print]. 30377729

[238] Gómez-Andrés D Dabaj I Mompoint D Hankiewicz K Azzi V Ioos C. Pediatric laminopathies: whole-body magnetic resonance imaging fingerprint and comparison with Sepn1 myopathy. Muscle Nerve (2016) 54:192–202. 10.1002/mus.25018 26670690

[239] Malattia C Damasio MB Madeo A Pistorio A Providenti A Pederzoli S. Whole-body MRI in the assessment of disease activity in juvenile dermatomyositis. Ann Rheum Dis. (2014) 73:1083–90. 10.1136/annrheumdis-2012-202915 23636654

